# High Diversity of *Cytospora* Associated With Canker and Dieback of Rosaceae in China, With 10 New Species Described

**DOI:** 10.3389/fpls.2020.00690

**Published:** 2020-07-03

**Authors:** Meng Pan, Haiyan Zhu, Guido Bonthond, Chengming Tian, Xinlei Fan

**Affiliations:** ^1^Key Laboratory for Silviculture and Conservation of Ministry of Education, Beijing Forestry University, Beijing, China; ^2^State Key Laboratory of Mycology, Institute of Microbiology, Chinese Academy of Sciences, Beijing, China; ^3^College of Life Sciences, University of Chinese Academy of Sciences, Beijing, China; ^4^GEOMAR Helmholtz Centre for Ocean Research Kiel, Kiel, Germany

**Keywords:** Cytosporaceae, Diaporthales, phylogeny, taxonomy, 10 new taxa

## Abstract

Cytospora canker is a destructive disease of numerous hosts and causes serious economic losses with a worldwide distribution. Identification of *Cytospora* species is difficult due to insufficient phylogenetic understanding and overlapped morphological characteristics. In this study, we provide an assessment of 23 *Cytospora* spp., which covered nine genera of Rosaceae, and focus on 13 species associated with symptomatic branch or twig canker and dieback disease in China. Through morphological observation and multilocus phylogeny of internal transcribed spacer (ITS), large nuclear ribosomal RNA subunit (LSU), actin (*act*), RNA polymerase II subunit (*rpb2*), translation elongation factor 1-α (*tef1-*α), and beta-tubulin (*tub2*) gene regions, the results indicate 13 distinct lineages with high branch support. These include 10 new *Cytospora* species, i.e., *C. cinnamomea*, *C. cotoneastricola*, *C. mali-spectabilis*, *C. ochracea*, *C. olivacea*, *C. pruni-mume*, *C. rosicola*, *C. sorbina*, *C. tibetensis*, and *C. xinjiangensis* and three known taxa including *Cytospora erumpens*, *C. leucostoma*, and *C. parasitica*. This study provides an initial understanding of the taxonomy of *Cytospora* associated with canker and dieback disease of Rosaceae in China.

## Introduction

Many species of Rosaceae plants have economic value, which are important parts of the greening of urban parks and widely cultivated around the world. Many species are also famous fresh and dried fruits with excellent varieties. However, most Rosaceae plants are under serious disease of dieback and stem canker caused by *Cytospora* species, which have contributed to a severe reduction in yield and quality. A study to clarify the pathogens causing stem canker and dieback of Rosaceae plants is urgently needed.

*Cytospora* was first introduced by Ehrenberg (1818), which is one of the most important pathogenic fungi of hardwoods and coniferous trees in the world (Adams et al., 2005; Fan et al., 2020). About 150 species epithets of *Cytospora* are associated with dieback and stem canker on over 130 species of woody hosts (Spielman, 1985; Adams et al., 2005; Kirk et al., 2008; Fan et al., 2020). Over 660 species epithets of *Cytospora* have been described in Index Fungorum (2020). However, most of these were regarded as synonyms, and most descriptions were unable to identify them accurately (Adams et al., 2005). *Leucostoma*, *Valsa*, *Valsella*, and *Valseutypella* have been treated as the synonyms of *Cytospora* (Rossman et al., 2015). The traditional identification of *Cytospora* species was based heavily on their host affiliations; nevertheless, a single species of *Cytospora* may occur on a different host, and more than one *Cytospora* species may be isolated from a single host (Adams et al., 2005; Wang et al., 2011; Ariyawansa et al., 2015; Fan et al., 2015a, b; Hyde et al., 2016; Lawrence et al., 2018). Accurate identification needs additional informative morphological observation and multilocus phylogeny to test the relationship among species (Adams and Taylor, 1993; Harrington and Rizzo, 1999; Adams et al., 2002). Adams et al. (2005) introduced 28 species of *Cytospora* from *Eucalyptus* by morphology and phylogeny using ITS sequence. A total of 144 strains from Iran represented 20 species of *Cytospora* based on ITS phylogeny (Fotouhifar et al., 2010). Later, many species were described based on multilocus phylogeny in recent studies (Fan et al., 2014a,b, 2015a,b; Yang et al., 2015; Lawrence et al., 2017, 2018; Zhu et al., 2018, 2020; Jiang et al., 2020; Shang et al., 2020). Fan et al. (2020) provided an assessment of 52 species of *Cytospora* in China using a six-locus phylogeny [internal transcribed spacer (ITS), large nuclear ribosomal RNA subunit (LSU), actin (*act*), RNA polymerase II subunit (*rpb2*), translation elongation factor 1-α (*tef1-*α), and beta-tubulin (*tub2*)]. However, most boundaries of known *Cytospora* species are tentative and indistinct due to the overlapped morphological characteristics, poor condition of multilocus phylogeny (only ITS is available for most species), and the shortage of fresh collected specimens. Thus, a geography- or host-centered strategy to define species of *Cytospora* using multiphase approaches has been proposed (Fan et al., 2020).

In this study, a total of 29 strains of *Cytospora* were isolated from symptomatic hosts of Rosaceae in China. The objectives were to (1) define the species of *Cytospora* associated with canker and dieback disease of Rosaceae, with illustrations and descriptions; (2) supplement a multi-gene DNA dataset of *Cytospora*, including ITS, LSU, *act*, *rpb2*, *tef1-*α, and *tub2*.

## Materials and Methods

### Sample Collection and Isolation

**FIGURE 1:**
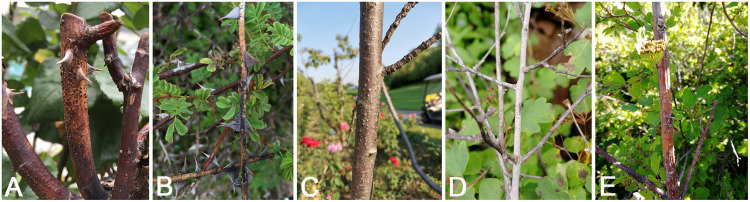
Disease symptoms associated with *Cytospora* species. **(A,B)** *Rosa* sp. **(C)** *Prunus serrulata*. **(D,E)** *Spiraea salicifolia*.

**FIGURE 2:**
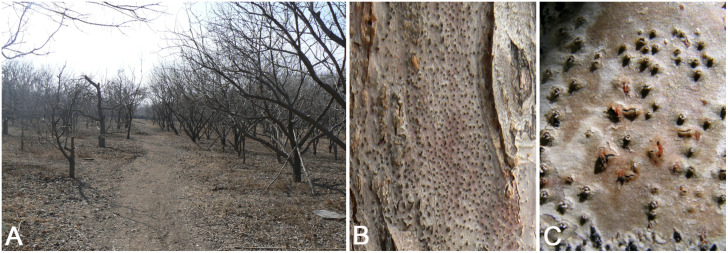
Disease symptoms associated with *Cytospora* species on *Malus* sp. **(A)** Death of the apple trees caused by *Cytospora* in the orchards. **(B,C)** Conidiomata on a naturally infected stem in the field.

Fresh specimens of Cytospora canker disease symptoms were collected from infected branches or twigs of five host genera of Rosaceae (11 host species) during collecting trips in China (Supplementary Table S1). The symptoms of Cytospora dieback disease included wilting and killing of twigs and branches, which commenced at the tips and progressed downward to the larger branches, inducing wood lesions and canker formation. Cytospora canker disease represents slightly sunken and discolored areas in the bark, diseased inner bark, and the bark above the infected cambium may appear yellow, brown, reddish brown, gray, or black, becoming watery and odorous as the tissues deteriorate. Several prominent dark sporocarps immersed in the bark, erumpent through the surface of bark when mature (Figures 1, 2). The occurrence of canker diseases of *Cytospora* in Rosaceae is widespread, which could cause a large area of death in several apple orchards (Figure 2). A total of 29 strains were isolated by removing a mucoid spore mass from conidiomata and/or ascomata, spreading the suspension on the surface of 1.8% potato dextrose agar (PDA) in a Petri dish, and incubating at 25°C for up to 24 h. Single-germinating conidia were transferred onto fresh PDA plates. All specimens are deposited at the Museum of the Beijing Forestry University (BJFC) and the working Collection of X.L. Fan (CF) housed at the Beijing Forestry University. Living cultures are deposited at the China Forestry Culture Collection Centre (CFCC).

### Morphology

Observation and description of *Cytospora* species were based on morphological characteristics of the fruiting bodies produced on infected host materials including arrangement and size of stromata; presence and absence of a conceptacle; size, color, and shape of discs; and number and diameter of ostioles per disc. The morphological characteristics were determined under a Leica stereomicroscope (M205). Micro-morphological observations include size and shape of conidiophores and conidia determined under a Nikon Eclipse 80i microscope. Over 30 conidiomata were sectioned, and 50 conidia were selected randomly for measurement. Incubation was done on PDA at 25°C in darkness, and colony diameters and colors were recorded and described after 1 or 2 weeks according to the color charts of Rayner (1970). Adobe Bridge CS v.6 and Adobe Photoshop CS v.5 were used for the manual editing. Taxonomic novelties were deposited in MycoBank (Crous et al., 2004).

### DNA Extraction and PCR Amplification

Genomic DNA was extracted from mycelium cultured on PDA with cellophane for 3 days using the modified CTAB method (Doyle and Doyle, 1990). The extracted DNA was estimated visually by electrophoresis in 1% agarose gels, and band intensity was compared with a DNA maker, 1 kbp (Takara Biotech). The qualities of DNA were measured with NanoDrop^TM^ 2000 (Thermo, United States). The internal transcribed spacer (ITS) region was amplified with primers ITS1 and ITS4 (White et al., 1990). The large nuclear ribosomal RNA subunit (LSU) region was amplified with primers LROR and LR7 (Vilgalys and Hester, 1990). The actin (*act*) region was amplified with primers ACT-512F and ACT-783R (Carbone and Kohn, 1999). The RNA polymerase II subunit (*rpb2*) region was amplified with primers RPB2-5F and fRPB2-7cR (Liu et al., 1999). The translation elongation factor 1-α (*tef1-*α) gene was amplified with primers EF-688F and EF-1251R (Carbone and Kohn, 1999). The beta-tubulin (*tub2*) gene was amplified with Bt-2a and Bt-2b (Glass and Donaldson, 1995). The PCR amplicons were electrophoresed in 2% agarose gels. DNA sequencing was carried out using an ABI PRISM^®^ 3730XL DNA Analyzer with BigDye^®^ Terminater Kit v.3.1 (Invitrogen) at the Shanghai Invitrogen Biological Technology Company Limited (Beijing, China). DNA sequences generated by the forward and reverse primers were used to obtain consensus sequences using Seqman v.9.0.4 (DNASTAR Inc., Madison, WI, United States).

### Phylogenetic Analyses

To infer a preliminary phylogenetic relationship for the new sequences, the first alignment based on ITS sequence data was performed using MAFFT v.6 (Katoh and Standley, 2013) and edited manually using MEGA v.6.0 (Tamura et al., 2013). Some characters were excluded from both ends of the alignments to approximate the size of our sequences to those included in the dataset. A second alignment was performed based on a combined six concatenate sequences (ITS, LSU, *act*, *rpb2*, *tef1-*α, and *tub2*). For individual datasets, sequences were aligned using MAFFT v.6 and edited manually using MEGA v.6.0 and some characters were excluded from both ends of the alignments. A partition homogeneity test (PHT) with heuristic search and 1,000 homogeneity replicates was performed using PAUP v.4.0b10 to test the discrepancy among the six-gene dataset in reconstructing phylogenetic trees. The sequences of *Diaporthe vaccinii* (CBS 160.32) was included as outgroup in all analyses. The phylogenetic analyses for all the datasets were run using PAUP v.4.0b10 for maximum parsimony (MP) (Swofford, 2003), MrBayes v.3.1.2 for Bayesian inference (BI) (Ronquist and Huelsenbeck, 2003), and RAxML-NG v.0.9.0 for maximum likelihood (ML) (Kozlov et al., 2019). Trees were visualized using FigTree v.1.3.1 (Rambaut and Drummond, 2010).

MP analysis was performed using a heuristic search (1,000 bootstraps) (Hillis and Bull, 1993), with random sequence addition as option to stepwise addition (1,000 replicates and one tree held at each addition step), and maxtrees limited to 200 by replicate. The tree bisection and reconnection (TBR) algorithm was selected (Swofford, 2003). The branches of zero length were collapsed using the command minbrlen, and all equally most parsimonious trees were saved. Other parsimony scores such as tree length (TL), consistency index (CI), retention index (RI), and rescaled consistency (RC) were calculated to describe tree statistics (Swofford, 2003). The branch supports of MP were evaluated with a bootstrapping (BS) method of 1,000 replicates (Hillis and Bull, 1993). For ML and BI analyses, the best-fit evolutionary models for each partitioned locus were estimated by MrModeltest v.2.3 following the Akaike Information Criterion (AIC) (Posada and Crandall, 1998). ML analysis was performed with RAxML-NG^1^ (Kozlov et al., 2019). The bootstrap was used with 100 replicates and the appropriate models for each gene. BI analysis was done by a Markov chain Monte Carlo (MCMC) algorithm with Bayesian posterior probabilities (BPP) (Rannala and Yang, 1996). Two MCMC chains were run from random trees for 10 million generations, and trees were sampled each 100th generation. The first 25% of the trees were discarded as the burn-in phase of each analysis; branches with significant BPP were calculated to assess the remaining trees (Rannala and Yang, 1996). Phylograms are shown using Figtree v.1.3.1 (Rambaut and Drummond, 2010). All sequences from this study data were deposited in GenBank. The ITS and multi-gene sequence alignment files were deposited in TreeBASE^2^ (accession number: S25903).

## Results

A total of 29 *Cytospora* isolates from Rosaceae hosts were collected in China. Following alignment, the ITS sequence data comprised 248 *Cytospora* in group taxa with a total of 629 characters including gaps, of which 365 characters were constant, 63 variable characters were parsimony uninformative, and 201 characters were variable and parsimony informative. A heuristic search generated 200 equally parsimonious trees each with similar clade topologies, and one of which is presented in Supplementary Figure S1 (TL = 1,223, CI = 0.343, RI = 0.855, RC = 0.294). ML and Bayesian results do not significantly differ from the MP tree.

**FIGURE 3:**
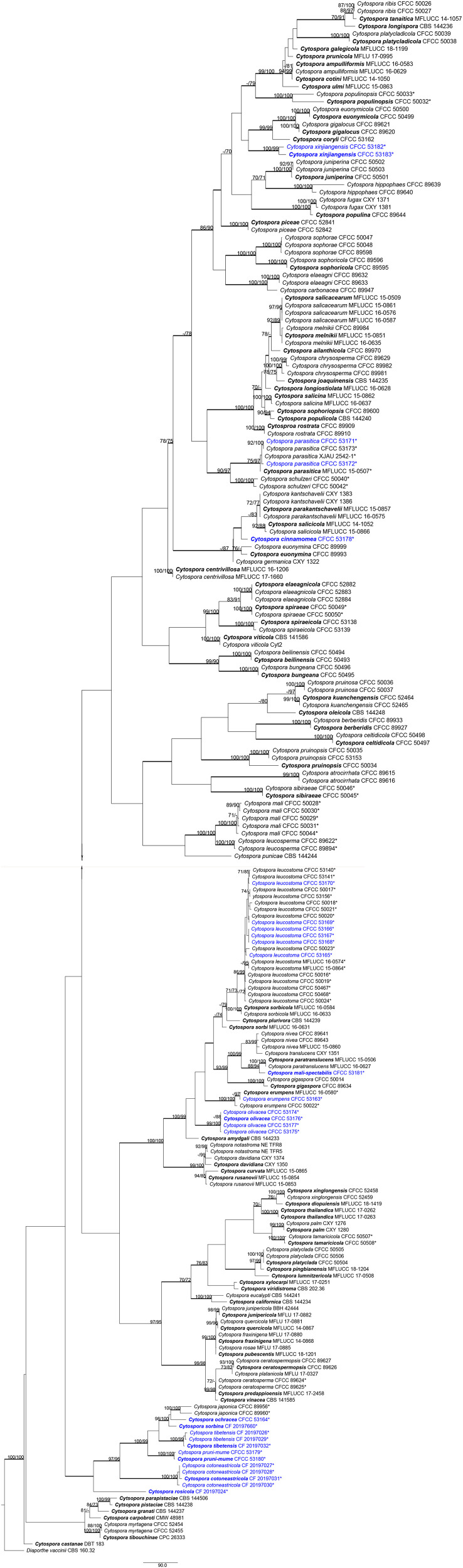
Phylogram of *Cytospora* based on combined internal transcribed spacer (ITS), large nuclear ribosomal RNA subunit (LSU), actin (*act*), RNA polymerase II subunit (*rpb2*), translation elongation factor 1-α (*tef1-*α), and beta-tubulin (*tub2*) genes. Maximum parsimony (MP) and maximum likelihood (ML) bootstrap support values above 70% are shown at the first and second positions. Thickened branches represent posterior probabilities above 0.95 from Bayesian inference (BI). Ex-type strains are in bold. Strains in current study are in blue. All the *Cytospora* species listed from Rosaceae plants in China are marked with *.

To clarify the phylogenetic position of these *Cytospora* species, a multi-locus analysis (ITS, LSU, *act*, *rpb2*, *tef1-*α, and *tub2*) is presented in Figure 3. The final analysis combined sequence data of six genes composed of 218 *Cytospora* ingroup taxa with a total of 3,712 characters including gaps, of which 2,037 characters were constant, 202 variable characters were parsimony uninformative, and 1,473 characters were variable and parsimony informative. MP analysis generated 200 equally parsimonious trees each with similar clade topologies, and one of which is presented in Figure 3 (TL = 9,782, CI = 0.303, RI = 0.803, RC = 0.244). For ML and BI analyses, the best-fit model of nucleotide evolution was deduced on the AIC (ITS and LSU: GTR, *act*: TVM, *rpb2* and *tef1-*α: TrN, and *tub*: HKY). ML method and Bayesian analyses were in agreement and no difference from the MP tree. The MP bootstrap supports (MP-BS) and ML bootstrap (ML-BS) equal to or above 70% were shown in branches in Figure 3. The branches with significant Bayesian posterior probabilities (BPP) equal to or above 0.95 are thickened in the phylograms.

The current 29 strains clustered in 13 clades were equivalent to 13 *Cytospora* species in Figure 3, including three known species (*Cytospora erumpens*, *C. leucostoma*, and *C. parasitica*) and 10 new clades, distinct from all known taxa, are herein described as *C. cinnamomea*, *C. cotoneastricola*, *C. mali-spectabilis*, *C. ochracea*, *C. olivacea*, *C. pruni-mume*, *C. rosicola*, *C. sorbina*, *C. tibetensis*, and *C. xinjiangensis.* All detailed descriptions and notes are below.

### Taxonomy

**FIGURE 4:**
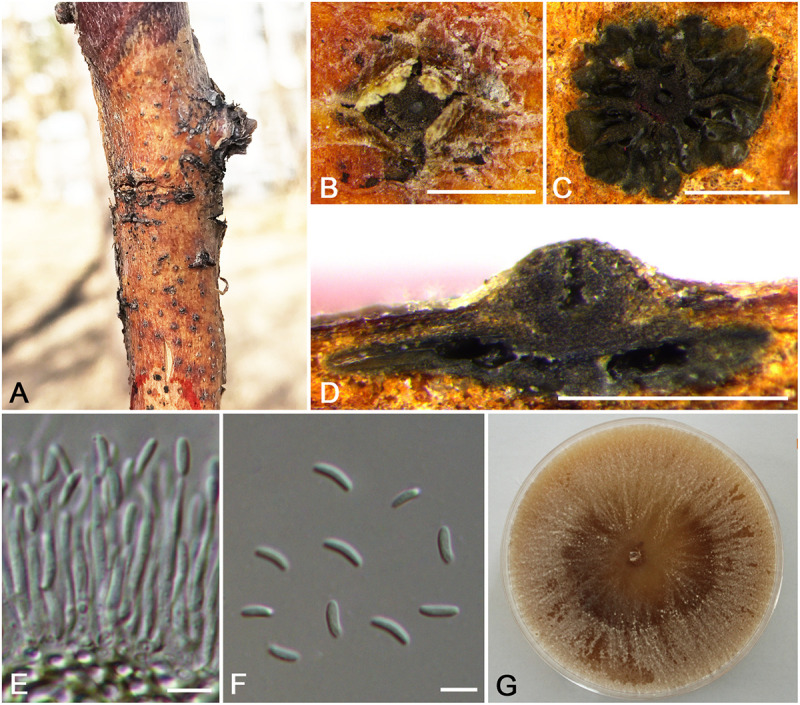
Morphology of *Cytospora cinnamomea* from *Prunus armeniaca* (CF 20197654). **(A,B)** Habit of conidiomata on twig. **(C)** Transverse section of conidioma. **(D)** Longitudinal section through conidioma. **(E)** Conidiophores and conidiogenous cells. **(F)** Conidia. **(G)** Colonies on potato dextrose agar (PDA) for 2 weeks. Bars: **(B–D)** = 500 μm; **(E)** = 10 μm; **(F)** = 5 μm.

***Cytospora cinnamomea*** M. Pan & X.L. Fan, sp. nov. (Figure 4)

MycoBank MB 834846

*Typification*: China. Xinjiang Uygur Autonomous Region: Bole Mongol Autonomous Prefecture, 44°46′14.88′′N, 81°13′58.64′′E, from branches of *Prunus armeniaca*, 20 July 2017, *C.M. Tian* & *X.L. Fan* (**holotype** CF 20197654), ex-type living culture CFCC 53178.

*Etymology*: Named after the distinctive cinnamon culture color.

*Descriptions*: Symptoms appeared as circular to ovoid, brown or dark, raised, dehiscent lesions on the twigs or branches, with slightly discolored bark above the infected cambium. Asexual morph: Conidiomata pycnidial, immersed in bark, erumpent through the surface of bark when mature, erumpent, discoid, with multiple locules, 785–1,070 μm ($\bar{x}$ = 935 μm, *n* = 30) in diam. Conceptacle absent. Ectostromatic disc dark-brown, circular to ovoid, disc black, 280–560 μm ($\bar{x}$ = 425 μm, *n* = 30) in diam. Ostiole conspicuous, circular, gray to brown at the same level as the disc surface, 50–63 μm ($\bar{x}$ = 57 μm, *n* = 30) in diam. Locules numerous, irregular arrangement with individual walls. Conidiophores hyaline, unbranched at base or occasionally branched. Conidiogenous cells enteroblastic, phialidic, sub-cylindrical to cylindrical. Conidia hyaline, smooth-walled, elongate-allantoid, unicellular, (4.0–)4.5–6.0(–6.5) × 1–1.5 μm ($\bar{x}$ = = 5.3 × 1.3 μm, *n* = 50). Sexual morph: not observed.

*Culture characteristics*: Cultures on PDA are initially white, fast growing, and covering the 9-cm Petri dish after 3 days, becoming cinnamon to fawn after 30 days. The colonies are flat and with a uniform texture, conidiomata sparse and distributed irregularly on the medium surface.

*Habitat and distribution*: Known only on *Prunus armeniaca* from the type locality.

*Notes*: *Cytospora cinnamomea* is associated with canker disease of *Prunus armeniaca* in China. In the combined analysis, the most closely related species to *Cytospora cinnamomea* are *C. kantschavelii*, *C. parakantschavelii*, *C. salicicola*, and *C. euonymina* (Figure 3). *Cytospora cinnamomea* can be distinguished from *C. salicicola* and *C. euonymina* by the smaller conidia (4.5–6.0 × 1–1.5 vs. 6.9–7.6 × 1.4–1.5, 6.5–7.5 × 1.5–2 μm) (Saccardo, 1892; Norphanphoun et al., 2017). *Cytospora cinnamomea* is morphologically similar to *C. kantschavelii* and *C. parakantschavelii*, whereas the former species differs from them by having distinct discoid conidiomata and producing cinnamon color in culture media. As for its size of conidia, it is similar with *C. parakantschavelii* (5.3 × 1.3 vs. 5.3 × 1.4 μm), but different from *C. kantschavelii* (5.3 × 1.3 vs. 4–5 × 1.2 μm) (Gvritishvili, 1973; Norphanphoun et al., 2017).

**FIGURE 5:**
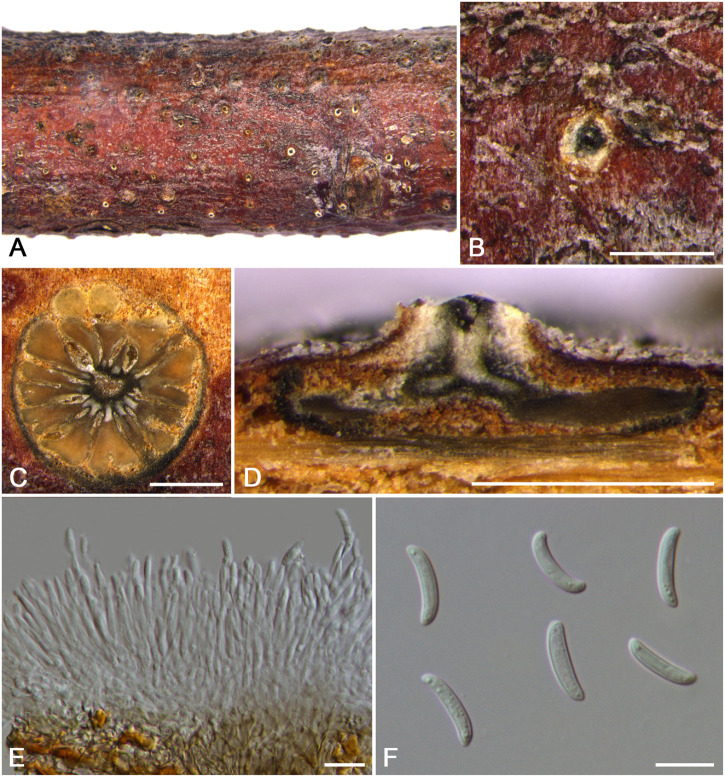
Morphology of *Cytospora cotoneastricola* from *Cotoneaster* sp. (CF 20197031). **(A,B)** Habit of conidiomata on twig. **(C)** Transverse section of conidioma. **(D)** Longitudinal section through conidioma. **(E)** Conidiophores and conidiogenous cells. **(F)** Conidia. Bars: **(B–D)** = 500 μm; **(E**,**F)** = 10 μm.

***Cytospora cotoneastricola*** M. Pan & X.L. Fan, sp. nov. (Figure 5)

MycoBank MB 834847

*Typification*: China. The Tibet Autonomous Region: Nyingchi City, Chayu County, Pineapple Village, 28°56′47.34′′N, 97°25′31.36′′E, from branches of *Cotoneaster* sp., 30 July 2016, *C.M. Tian* & *X.L. Fan* (**holotype** CF 20197031).

*Etymology*: Named after the host genus on which it was collected, *Cotoneaster* sp.

*Descriptions*: Symptoms appeared as circular to ovoid, yellow or light brown, raised lesions with a distinct black area at the center on the twigs or branches. Asexual morph: Conidiomata pycnidial, immersed in bark, erumpent through the surface of bark when mature, solitary, scattered, discoid to conical, with multi-locules, 1,200–1,480 μm ($\bar{x}$ = 1,350 μm, *n* = 30) in diam. Conceptacle conspicuous. Ectostromatic disc yellow to light brown, circular to ovoid, with one ostiole per disc, 165–310 μm ($\bar{x}$ = 240 μm, *n* = 30) in diam. Ostiole conspicuous, flask-shaped to conical, gray to black in the center of the disc at the same level as the disc surface, 110–165 μm ($\bar{x}$ = 131 μm, *n* = 30) in diam., a column lenticular tissue in the center. Locules dark brown, arranged circularly to triangularly or ellipse with independent walls, ovoid to spherical. Conidiophores hyaline, unbranched at base or occasionally branched. Conidiogenous cells enteroblastic, phialidic, sub-cylindrical to cylindrical. Conidia hyaline, smooth, allantoid, eguttulate, aseptate, thin-walled, (12.5–)13.0–14.0(–14.5) × 2.5–3 μm ($\bar{x}$ = 13.6 × 2.8 μm, *n* = 50). Sexual morph: not observed.

*Habitat and distribution*: Known on *Cotoneaster* sp. from the type locality and an additional locality in the Tibet Autonomous Region.

*Additional materials examined*: China. The Tibet Autonomous Region: Nyingchi City, Chayu County, Pineapple Village, 28°56′47.34′′N, 97°25′31.36′′E, from branches of *Cotoneaster* sp., 30 July 2016, *C.M. Tian* & *X.L. Fan* (CF 20197030); Nyingchi City, Chayu County, Pineapple Village, 28°56′47.33′′N, 97°25′31.44′′E, from branches of *Cotoneaster* sp., 30 July 2016, *C.M. Tian* & *X.L. Fan* (CF 20197027); ibid. CF 20197028.

*Notes*: *Cytospora cotoneastricola* is described as being associated with canker disease of *Cotoneaster* sp. in China. *Cytospora tibetensis* and *C. ochracea* are associated with the same host. Morphologically, *C. cotoneastricola* is distinguished from *C. tibetensis* by having multi-locules and larger size of conidia (13.0–14.0 × 2.5–3 vs. 5.0–5.5 × 1.5–2 μm). *Cytospora cotoneastricola* also has obvious central column and larger conidia than *C. ochracea* (13.5–14.0 × 2.5–3 vs. 8.5–9.0 × 1.5–2.5 μm). This species needs to be re-collected from *Cotoneaster* sp. in Tibet of China, as presently no living culture is available.

***Cytospora erumpens*** Norph. et al., Mycosphere 8: 64, 2017.

*Descriptions*: see Norphanphoun et al. (2017).

*Habitat and distribution*: Known on *Salix fragilis and Prunus padus*. This fungus has been reported from Russia and China.

*Material examined*: China. Xinjiang Uygur Autonomous Region: Ili Kazak Autonomous Prefecture, 46°01′17.82′′N, 82°45′08.94′′E, from branches of *Prunus padus*, 16 July 2017, *C.M. Tian* & *X.L. Fan* (CF 20197563), living culture CFCC 53163.

*Notes*: *Cytospora erumpens* was introduced to cause canker and dieback disease of *Salix* in Russia (Norphanphoun et al., 2017). Afterward, it was reported on *Prunus padus* in China (Fan et al., 2020). This fungus can be identified by its black-discoid conidiomata with long ostiolar necks, producing elongate-allantoid conidia (6.4–6.7 × 1.3–1.4 μm) (Norphanphoun et al., 2017). Combined morphology and the DNA sequence data of our strain, which was collected from dead branches of *Prunus padus* belongs to this species.

***Cytospora leucostoma*** (Pers.) Sacc., Michelia 2: 264, 1881.

*Synonyms*: *Sphaeria leucostoma* Pers., Ann. Bot. 11: 23, 1794.

*Valsa leucostoma* (Pers.) Fr., Summa Veg. Scand., Section Post. (Stockholm): 411, 1849.

*Valsa persoonii* Nitschke, Pyrenomyc. Germ. 2: 222, 1870.

*Leucostoma persoonii* (Nitschke) Höhn., Mitt. Bot. Inst. Tech. Hochsch. Wien 5: 78, 1928.

*Cytospora donetzica* Norphanph et al., Mycosphere 8: 62, 2017.

*Valsa ambiens* (Pers.) Fr., Summa Veg. Scand., Sectio Post. (Stockholm): 412, 1849.

*Cytospora ambiens* Sacc., Michelia 1(5): 519, 1879.

*Descriptions*: see Fan et al. (2020).

*Habitat and distribution*: Known from mainly Roseaceae, especially Prunoideae. This fungus has been reported around the world.

*Materials examined*: China. Xinjiang Uygur Autonomous Region: Bole Mongol Autonomous Prefecture, 44°46′12.65′′N, 81°14′02.62′′E, from branches of *Sorbus tianschanica*, 20 July 2017, *C.M. Tian* & *X.L. Fan* (CF 20197672), living culture CFCC 53165; Ili Kazak Autonomous Prefecture, 44°27′39.68′′N, 80°21′12.86′′E, from branches of *Prunus armeniaca*, 22 July 2017, *C.M. Tian* & *X.L. Fan* (CF 20197710), living culture CFCC 53166; ibid. CF 20197711, living culture CFCC 53167; Ili Kazak Autonomous Prefecture, 46°20′55.49′′N, 83°53′55.94′E, from branches of *Prunus pseudocerasus*, 14 July 2017, *C.M. Tian* & *X.L. Fan* (CF 20197513), living culture CFCC 53168. Beijing: Songshan National Nature, 40°30′25.07′′N, 115°48′44.36′′E, from branches of *Prunus persica*, 20 July 2017, *C.M. Tian* & *X.L. Fan* (CF 20191281), living culture CFCC 53169; ibid. CF 20191284, living culture CFCC 53170.

*Notes*: *Cytospora leucostoma* is a common species associated with stem canker diseases of woody plants of Rosaceae in China (Fan et al., 2020). This species has obvious black conceptacle, numerous locules, which were subdivided frequently by invaginations with independent walls, and hyaline, allantoid, aseptate conidia with the size of 4.5–5.5 × 1–1.5 μm. In a recent study, *Cytospora donetzica* has been treated as the synonym of *C. leucostoma* based on Fan et al. (2020).

**FIGURE 6:**
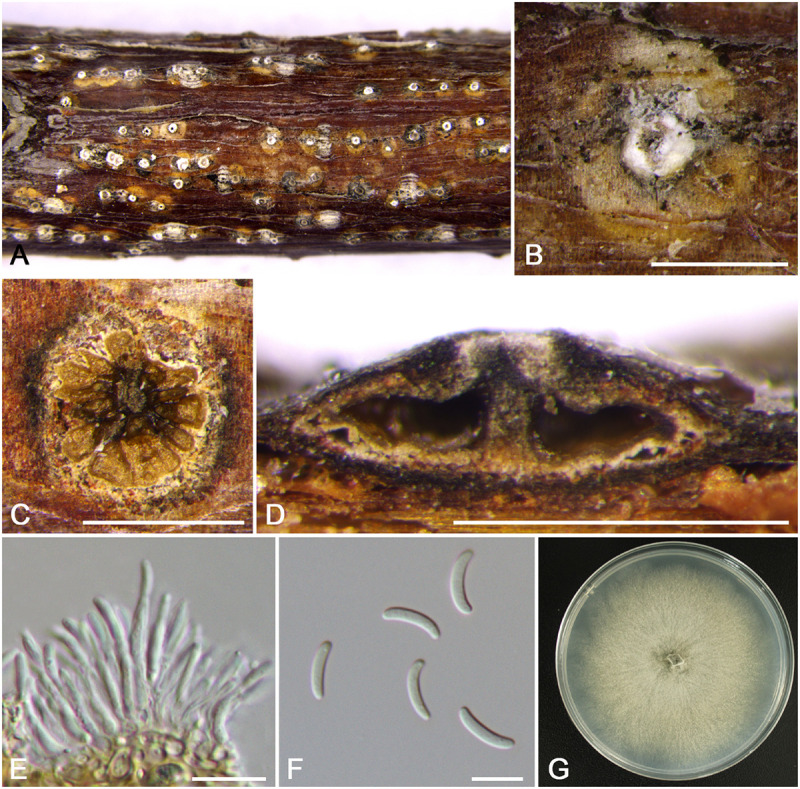
Morphology of *Cytospora mali-spectabilis* from *Malus spectabilis* ‘Royalty’ (CF 20197665). **(A,B)** Habit of conidiomata on twig. **(C)** Transverse section of conidioma. **(D)** Longitudinal section through conidioma. **(E)** Conidiophores and conidiogenous cells. **(F)** Conidia. **(G)** Colonies on PDA for 2 weeks. Bars: **(B–D)** = 500 μm; **(E,F)** = 10 μm.

***Cytospora mali-spectabilis*** M. Pan & X.L. Fan, sp. nov. (Figure 6)

MycoBank MB 834848

*Typification*: China. Xinjiang Uygur Autonomous Region: Bole Mongol Autonomous Prefecture, 44°46′10.79′′N, 81°13′56.60′′E, from branches of *Malus spectabilis* ‘Royalty’, 20 July 2017, *C.M. Tian* & *X.L. Fan* (**holotype** CF 20197665), ex-type living culture CFCC 53181.

*Etymology*: Named after the host genus on which it was collected, *Malus spectabilis* ‘Royalty’.

*Descriptions*: Symptoms appeared as circular, white or ashen, raised lesions with a distinct brown or dark area at the center on the twigs or branches, with discolored bark above the infected cambium. Asexual morph: Conidiomata pycnidial, immersed in bark, erumpent through the surface of bark when mature, erumpent, discoid to conical, 580–675 μm ($\bar{x}$ = 630 μm, *n* = 30) in diam., with multi-locule, a column lenticular tissue in the center. Conceptacle absent. Ectostromatic disc white to brown, circular to ovoid, disc dark yellow to brown, 240–350 μm ($\bar{x}$ = 295 μm, *n* = 30) in diam. Ostiole conspicuous, circular to ovoid, gray to black at the same level as the disc surface, 60–84 μm ($\bar{x}$ = 69 μm, *n* = 30) in diam. Locules complex with wild shapes, subdivided by invaginations with common walls. Conidiophores hyaline, unbranched at base or occasionally branched. Conidiogenous cells enteroblastic, phialidic, sub-cylindrical to cylindrical. Conidia hyaline, unicellular, eguttulate, elongate-allantoid, (8.0–)9.0–10.0(–11.0) × 1.5–2 μm ($\bar{x}$ = 9.5 × 1.8 μm, *n* = 50). Sexual morph: not observed.

*Culture characteristics*: Cultures on PDA are initially white, growing up to 6.5 cm after 3 days, entirely covering the 9-cm Petri dish and becoming buff after 7 days. The colonies ultimately are gray olivaceous and flat with a uniform texture. Conidiomata are randomly distributed on medium surface.

*Habitat and distribution*: Known only on *Malus spectabilis* ‘Royalty’ from the type locality.

*Notes*: *Cytospora mali-spectabilis* is associated with canker disease of *Malus spectabilis* ‘Royalty’. In the phylogenetic analyses, *C. mali-spectabilis* clusters with *C. paratranslucens* and *C. nivea* with high bootstrap support (MP/ML/BI = 100/99/1). However, it can be distinguished from *C. paratranslucens* and *C. nivea* by larger conidia (9.0–10.0 × 1.5–2 vs. 6.5–7.3 × 1.3–1.5, 7.4–8.8 × 1.5–1.6 μm), and the distinct central column of conidia (Adams et al., 2006; Norphanphoun et al., 2017). Furthermore, *C. mali-spectabilis* has multiloculate conidiomata sharing a smaller single ostiole (60–84 vs. 70–150 μm) than *C. paratranslucens* (Norphanphoun et al., 2017). *Cytospora mali-spectabilis* has absent conceptacle, whereas *C. nivea* owned black conceptacle surrounding the asexual stroma, usually presenting a huge black ectostromatic disc on the bark surface (Adams et al., 2005; Fan et al., 2014b).

**FIGURE 7:**
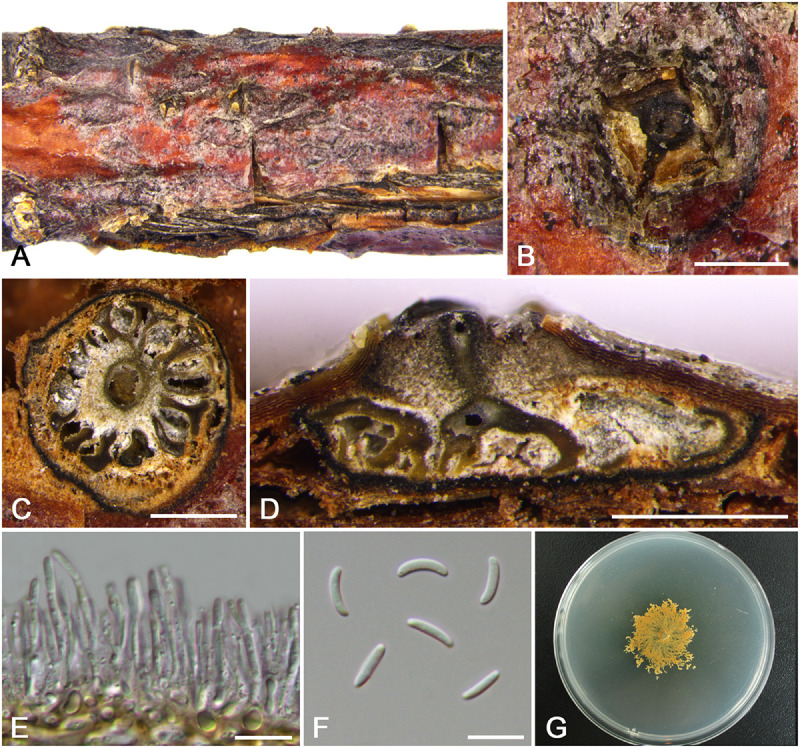
Morphology of *Cytospora ochracea* from *Cotoneaster* sp. (CF 20197684). **(A,B)** Habit of conidiomata on twig. **(C)** Transverse section of conidioma. **(D)** Longitudinal section through conidioma. **(E)** Conidiophores and conidiogenous cells. **(F)** Conidia. **(G)** Colonies on PDA for 2 weeks. Bars: **(B–D)** = 500 μm; **(E,F)** = 10 μm.

***Cytospora ochracea*** M. Pan & X.L. Fan, sp. nov. (Figure 7)

MycoBank MB 834849

*Typification*: China. Xinjiang Uygur Autonomous Region: Bole Mongol Autonomous Prefecture, 44°46′26.42′′N, 81°11′19.28′′E, from branches of *Cotoneaster* sp., 20 July 2017, *C.M. Tian* & *X.L. Fan* (**holotype** CF 20197684), ex-type living culture CFCC 53164.

*Etymology*: Named after the distinctive ochreous culture color.

*Descriptions*: Symptoms appeared as elongate, circular to ovoid, raised, dehiscent lesions surrounded by a black circle on the twigs or branches, with slightly discolored bark above the infected cambium. Sometimes lesions may split along the canker margin. Asexual morph: Conidiomata pycnidial, immersed in bark, erumpent through the surface of bark when mature, erumpent, discoid to conical, 1,150–1,325 μm ($\bar{x}$ = 1,230 μm, *n* = 30) in diam. Conceptacle dark. Ectostromatic disc dark brown, circular to ovoid, disc gray white to dark brown, 630–850 μm ($\bar{x}$ = 740 μm, *n* = 30) in diam. Ostiole conspicuous, circular, dark-brown to black, 180–260 μm ($\bar{x}$ = 235 μm, *n* = 30) in diam. Locules numerous, subdivided frequently by invaginations, irregular arrangement with individual walls. Conidiophores hyaline, unbranched at base or occasionally branched. Conidiogenous cells enteroblastic, phialidic, sub-cylindrical to cylindrical. Conidia hyaline, eguttulate, smooth-walled, elongate-allantoid, aseptate, (7.5–)8.5–9.0(–10.0) × 1.5–2.5 μm ($\bar{x}$ = 8.8 × 1.9 μm, *n* = 50). Sexual morph: not observed.

*Culture characteristics*: Cultures on PDA are initially white, growing up to 5.5 cm after 3 days, becoming ochreous in center after 7 days, deepened in later stage gradually. Colonies are tight, thin with a uniform texture, lacking aerial mycelium. Conidiomata are randomly distributed on medium surface.

*Habitat and distribution*: Known only on *Cotoneaster* sp. from the type locality.

*Notes*: *Cytospora ochracea* is associated with canker disease of *Cotoneaster* sp. In the phylogenetic analyses, *C. ochracea* clusters with *C. japonica* and *C. sorbina* with high bootstrap support (MP/ML/BI = 98/100/1). However, it can be distinguished from *C. japonica* and *C. sorbina* by larger conidia (8.5–9.0 × 1.5–2.5 vs. 6.5–8.5 × 1.5–2, 4.5–5.5 × 1–1.5 μm) (Fan et al., 2020). Furthermore, *C. ochracea* has multiloculate conidiomata with individual walls, whereas *C. sorbina* owned locules with the common walls. Also *C. ochracea* differs from *C. japonica* in culture characteristics of its color and growth rate.

**FIGURE 8:**
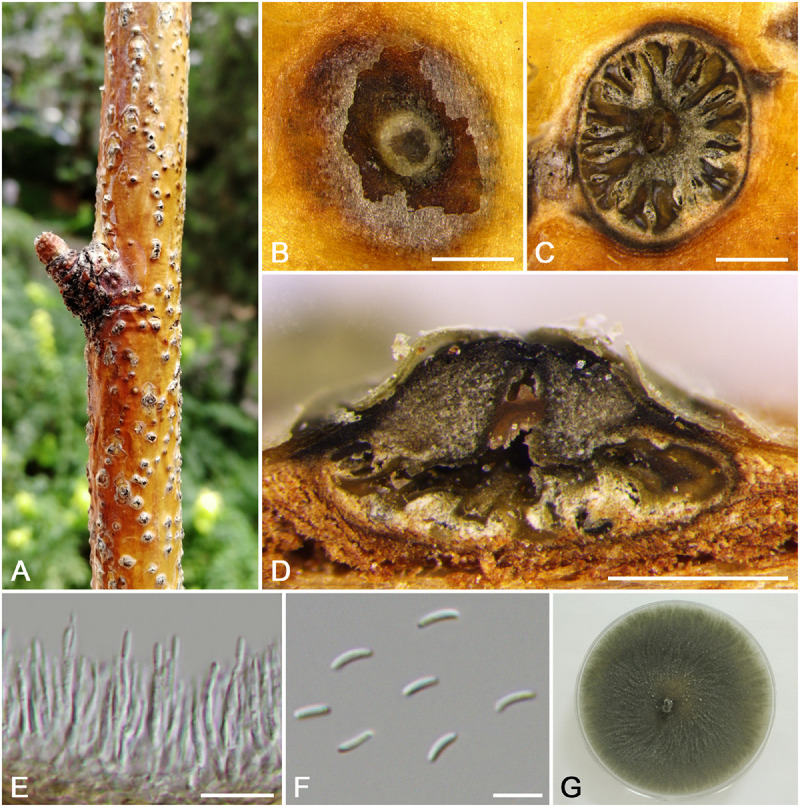
Morphology of *Cytospora olivacea* from *Sorbus tianschanica* (CF 20197670). **(A,B)** Habit of conidiomata on twig. **(C)** Transverse section of conidioma. **(D)** Longitudinal section through conidioma. **(E)** Conidiophores and conidiogenous cells. **(F)** Conidia. **(G)** Colonies on PDA for 2 weeks. Bars: **(B–D)** = 500 μm; **(E)** = 10 μm; **(F)** = 5 μm.

***Cytospora olivacea*** M. Pan & X.L. Fan, sp. nov. (Figure 8)

MycoBank MB 834850

*Typification*: China. Xinjiang Uygur Autonomous Region: Bole Mongol Autonomous Prefecture, 44°46′10.79′′N, 81°13′56.60′′E, from branches of *Sorbus tianschanica*, 20 July 2017, *C.M. Tian* & *X.L. Fan* (**holotype** CF 20197670), ex-type living culture CFCC 53176.

*Etymology*: Named after the distinctive olivaceous culture color.

*Descriptions*: Symptoms appeared as circular to ovoid, raised, dehiscent lesions on the twigs or branches, with slightly discolored bark above the infected cambium. Asexual morph: Conidiomata pycnidial, immersed in bark, erumpent through the surface of bark when mature, erumpent, flask shaped to conical, with multiple locules, 1,285–1,535 μm ($\bar{x}$ = 1,415 μm, *n* = 30) in diam. Conceptacle dark. Ectostromatic disc yellow to light brown, circular to ovoid, disc dark brown, 1,050–1,450 μm ($\bar{x}$ = 1,250 μm, *n* = 30) in diam. Ostiole conspicuous, circular, gray to brown at the same level as the disc surface, 330–465 μm ($\bar{x}$ = 400 μm, *n* = 30) in diam. Locules numerous, irregular arrangement with independent walls. Conidiophores hyaline, unbranched at base or occasionally branched. Conidiogenous cells enteroblastic, phialidic, sub-cylindrical to cylindrical. Conidia hyaline, smooth-walled, elongate-allantoid, unicellular, 4.0–5.0(–5.5) × 1–1.5 μm ($\bar{x}$ = 4.4 × 1.3 μm, *n* = 50). Sexual morph: not observed.

*Culture characteristics*: Cultures on PDA are initially white and become olivaceous buff, growing fast and entirely covering the 9-cm Petri dish after 3 days, becoming olivaceous gray and slight helical after 30 days. The colonies are flat and with a uniform texture. Conidiomata are randomly distributed on medium surface, extruding a pale white conidial mass.

*Habitat and distribution*: Known on *Cotoneaster* sp. and *Prunus* spp. from the type locality and an additional locality in Xinjiang Uygur Autonomous Region.

*Additional materials examined*: China. Xinjiang Uygur Autonomous Region: Ili Kazak Autonomous Prefecture, 45°54′18.17′′N, 83°20′45.35′′E, from branches of *Prunus dulcis*, 15 July 2017, *C.M. Tian* & *X.L. Fan* (CF 20197556), living culture CFCC 53175; Bole Mongol Autonomous Prefecture, 44°46′13.44″N, 81°13′58.72″E, from branches of *Prunus virginiana*, 18 July 2017, *C.M. Tian* & *X.L. Fan* (CF 20197601), living culture CFCC 53177; Bole Mongol Autonomous Prefecture, 44°46′13.78″N, 81°13′57.78″E, from branches of *Prunus cerasifera*, 20 July 2017, *C.M. Tian* & *X.L. Fan* (CF 20197652), living culture CFCC 53174.

*Notes*: *Cytospora olivacea* is associated with canker disease of *Prunus* spp. in China. It has multiple locules with black conceptacle, which is commonly discovered in *Cytospora* spp., while the molecular phylogenies show a clearly different position from all other strains included in this study. Therefore, we describe this species as novel based on morphology and combined sequence data of six genes.

**FIGURE 9:**
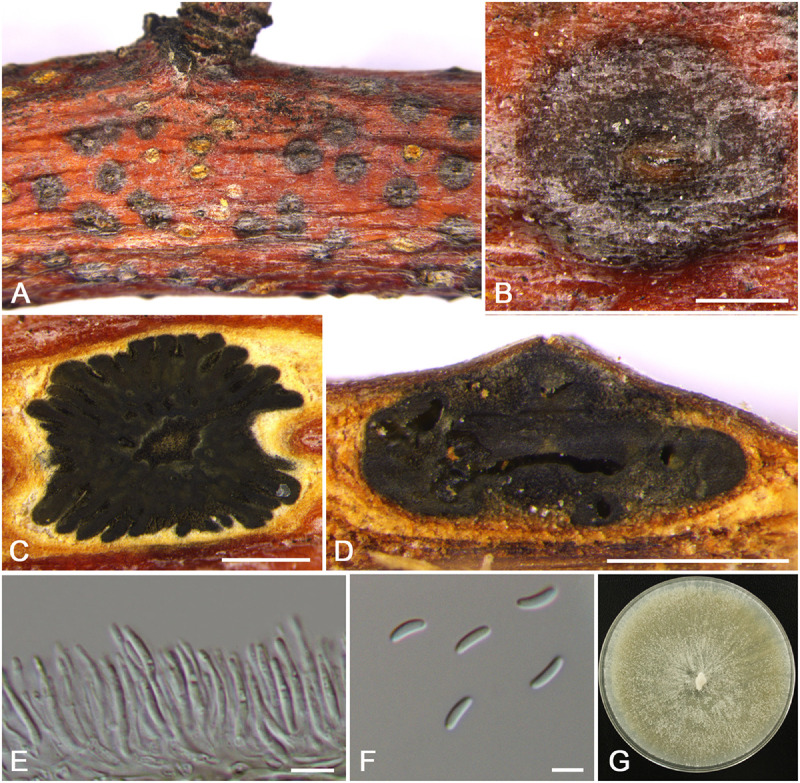
Morphology of *Cytospora parasitica* from *Malus pumila* (CF 20197714). **(A,B)** Habit of conidiomata on twig. **(C)** Transverse section of conidioma. **(D)** Longitudinal section through conidioma. **(E)** Conidiophores and conidiogenous cells. **(F)** Conidia. **(G)** Colonies on PDA for 2 weeks. Bars: **(B–D)** = 500 μm; **(E)** = 10 μm; **(F)** = 5 μm.

***Cytospora parasitica*** Norph. et al., Fung. Diversity 75: 146, 2015. (Figure 9)

*Descriptions*: Symptoms appeared as circular, dark brown or dark, slightly sunken, dehiscent lesions with a light brown area at the center on the twigs or branches, with discolored bark above the infected cambium. Asexual morph: Conidiomata pycnidial, immersed in bark, erumpent through the surface of bark when mature, erumpent, flask-shaped to conical, with multiple locules, 1,190–1,650 μm ($\bar{x}$ = 1,420 μm, *n* = 30) in diam. Conceptacle absent. Ectostromatic disc dark brown, circular to ovoid, disc gray to black, 1,190–1,480 μm ($\bar{x}$ = 1,310 μm, *n* = 30) in diam. Ostiole conspicuous, circular, dark brown to black, 160–210 μm ($\bar{x}$ = 185 μm, *n* = 30) in diam. Locules complex multi-loculed irregular arrangement subdivided frequently by invaginations, sharing common walls. Conidiophores hyaline, unbranched at base or occasionally branched. Conidiogenous cells enteroblastic, phialidic, sub-cylindrical to cylindrical. Conidia hyaline, smooth-walled, elongate-allantoid, aseptate, (5.0–)5.5–6.0(–6.5) × 1–2 μm ($\bar{x}$ = 5.7 × 1.5 μm, *n* = 50). Sexual morph: not observed.

*Culture characteristics*: Cultures on PDA are initially white, growing up to 8.5 cm after 3 days and entirely covering the 9-cm Petri dish after 7 days, becoming buff but white mostly. The colonies are flat with a uniform texture, becoming effuse on the surface, without aerial mycelium, conidiomata are randomly distributed on medium surface.

*Habitat and distribution*: Known from only *Malus* sp. in China and Russia.

*Materials examined*: China. Xinjiang Uygur Autonomous Region: Ili Kazak Autonomous Prefecture, 44°16′0.36″N, 80°24′55.53″E, from branches of *Malus pumila*, 22 July 2017, *C.M. Tian* & *X.L. Fan* (CF 20197714), living culture CFCC 53172; Ili Kazak Autonomous Prefecture, 45°56′49.20″N, 82°40′09.56″E, from branches of *Malus pumila*, 15 July 2017, *C.M. Tian* & *X.L. Fan* (CF 20197528), living culture CFCC 53171.

*Notes*: *Cytospora parasitica* was introduced by Ariyawansa et al. (2015) relating to canker disease of *Malus pumila.* Morphologically, our isolates are similar to *C. parasitica* in having multi-loculate pycnidial conidiomata, producing black area on bark, having smooth-walled, elongate-allantoid, aseptate conidia, whereas the size of our conidia differs from those isolates (5.5–6.0 × 1–2 vs. 6.5–8.0 × 1.3–1.5 μm) (Ariyawansa et al., 2015). Ma et al. (2018) reported this species from the same host plant *Malus pumila* in Xinjiang of China, which is similar with *C. parasitica* in the current study.

**FIGURE 10:**
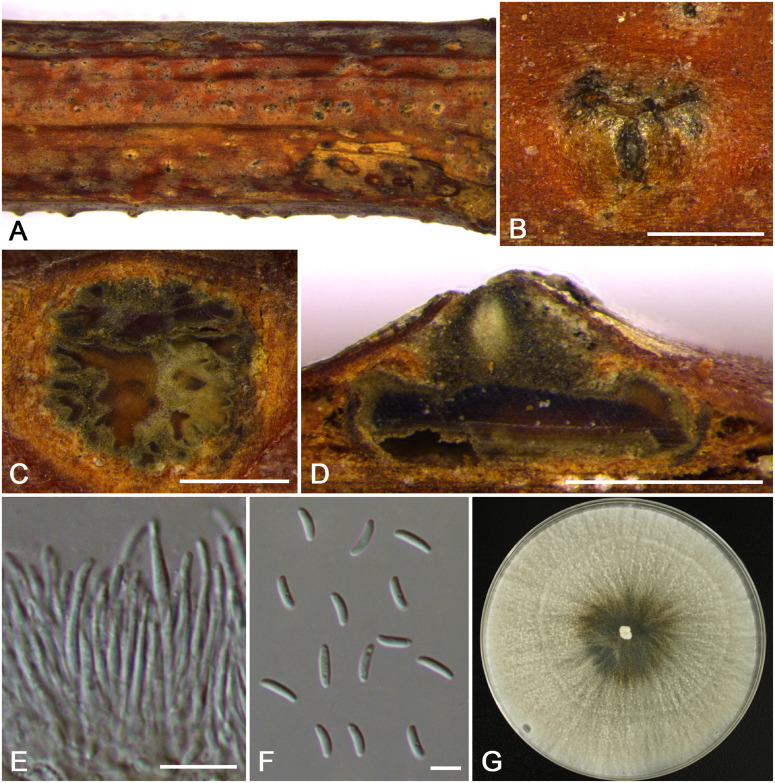
Morphology of *Cytospora pruni-mume* from *Prunus mume* (CF 20197515). **(A,B)** Habit of conidiomata on twig. **(C)** Transverse section of conidioma. **(D)** Longitudinal section through conidioma. **(E)** Conidiophores and conidiogenous cells. **(F)** Conidia. **(G)** Colonies on PDA for 2 weeks. Bars: **(B–D)** = 500 μm; **(E)** = 10 μm; **(F)** = 5 μm.

***Cytospora pruni-mume*** M. Pan & X.L. Fan, sp. nov. (Figure 10)

MycoBank MB 834851

*Typification*: China. Xinjiang Uygur Autonomous Region: Ili Kazak Autonomous Prefecture, 46°20′55.51″N, 83°53′55.96″E, from branches of *Prunus mume*, 14 July 2017, *C.M. Tian* & *X.L. Fan* (**holotype** CF 20197515), ex-type living culture CFCC 53180.

*Etymology*: Named after the host genus on which it was collected, *Prunus mume*.

*Descriptions*: Symptoms appeared as circular, raised, dehiscent lesions on the twigs or branches, with slightly discolored bark above the infected cambium. Asexual morph: Conidiomata pycnidial, immersed in bark, erumpent through the surface of bark when mature, erumpent, discoid, with multiple locules, 870–1,050 μm ($\bar{x}$ = 920 μm, *n* = 30) in diam. Conceptacle absent. Ectostromatic disc brown to dark brown, nearly hemispherical, disc dark brown to black, 510–780 μm ($\bar{x}$ = 630 μm, *n* = 30) in diam. Ostiole inconspicuous, circular to ovoid, gray to black at the same level as the disc surface. Locule multiple and complex, irregular distribution, subdivided by invaginations with common walls. Conidiophores hyaline, branched at base or occasionally not branched. Conidiogenous cells enteroblastic, phialidic, sub-cylindrical to cylindrical. Conidia hyaline, eguttulate, aseptate, smooth-walled, elongate-allantoid, (5.0–)5.5–6.5(–7.0) × 1.5–2 μm ($\bar{x}$ = 5.8 × 1.7 μm, *n* = 50). Sexual morph: not observed.

*Culture characteristics*: Cultures on PDA are initially white, growing fast and entirely covering the 9-cm Petri dish after 3 days, becoming pale yellow after 7 days. The colonies are flat with a uniform texture, conidiomata were randomly distributed on medium surface.

*Habitat and distribution*: Known on *Prunus mume* and *Prunus armeniaca* from the type locality.

*Additional material examined*: China. Xinjiang Uygur Autonomous Region: Ili Kazak Autonomous Prefecture, 46°20′54.76″N, 83°53′58.14″E, from branches of *Prunus armeniaca*, 14 July 2017, *C.M. Tian* & *X.L. Fan* (CF 20197512), living culture CFCC 53179.

*Notes*: *Cytospora pruni-mume* is associated with canker disease of *Prunus mume.* The molecular phylogenies show a position clearly distinct from all other strains included in this study (Figure 3). Therefore, we describe this species as a new species.

**FIGURE 11:**
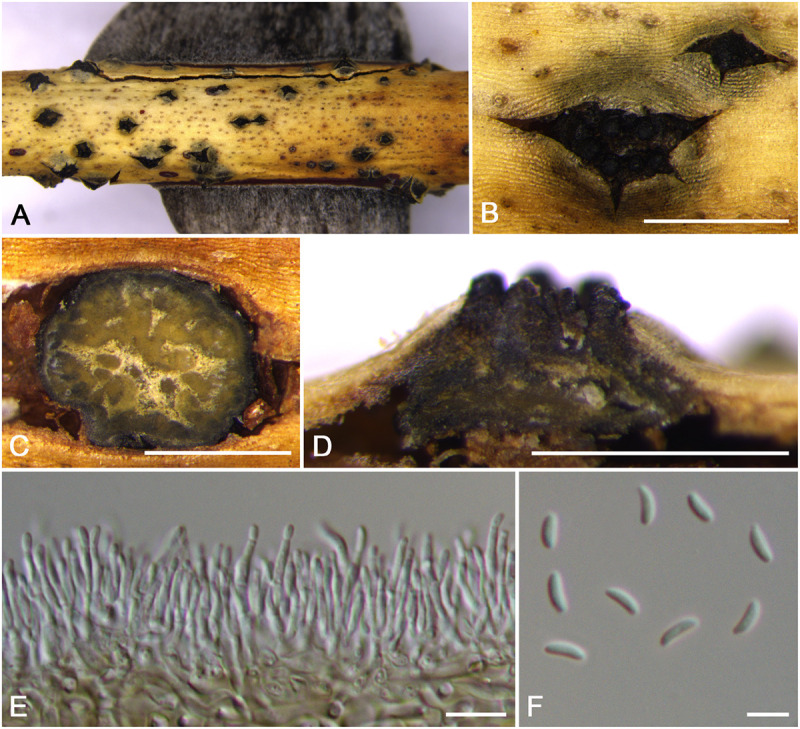
Morphology of *Cytospora rosicola* from *Rosa* sp. (CF 20197024). **(A,B)** Habit of conidiomata on twig. **(C)** Transverse section of conidioma. **(D)** Longitudinal section through conidioma. **(E)** Conidiophores and conidiogenous cells. **(F)** Conidia. Bars: **(B–D)** = 500 μm; **(E)** = 10 μm; **(F)** = 5 μm.

***Cytospora rosicola*** M. Pan & X.L. Fan, sp. nov. (Figure 11)

MycoBank MB 834853

*Typification*: China. The Tibet Autonomous Region: Nyingchi City, Nyingchi County, Nishi Village, 29°45′38.17″N, 94°16′16.71″E, from branches of *Rosa* sp., 23 July 2016, *C.M. Tian* & *X.L. Fan* (**holotype** CF 20197024).

*Etymology*: Named after the host genus on which it was collected, *Rosa* sp.

*Descriptions*: Symptoms appeared as circular to ovoid, gray or dark brown, raised, dehiscent lesions on the twigs or branches, with discolored bark above the infected cambium. Sometimes lesions may split along the canker margin. Asexual morph: Conidiomata pycnidial, immersed in bark, erumpent through the surface of bark when mature, solitary, scattered, breaking through the outer branch. Locules multiple, circular to ovoid, arranged vesicularly with common walls, 620–710 μm ($\bar{x}$ = 660 μm, *n* = 30) in diam. Conceptacle conspicuous. Ectostromatic disc brown to black, circular, disc dark brown, 380–660 μm ($\bar{x}$ = 520 μm, *n* = 30) in diam. Ostiole conspicuous, circular to ovoid, dark brown to black at the same level as the disc surface, 280–350 μm ($\bar{x}$ = 290 μm, *n* = 30) in diam. Conidiophores hyaline, branched at base or occasionally not branched. Conidiogenous cells enteroblastic, phialidic, sub-cylindrical to cylindrical. Conidia hyaline, allantoid, eguttulate, aseptate, thin-walled, (4.0–)4.5–5.0(–5.5) × 1–2 μm ($\bar{x}$ = 4.8 × 1.6 μm, *n* = 50). Sexual morph: not observed.

*Habitat and distribution*: Known only on *Rosa* sp. from the type locality.

*Notes*: *Cytospora* species associated with *Rosa* sp. were reported in previous studies such as *C. cincta* and *C. sacculus* (Zambettakis and Dzagania, 1986; Fotouhifar et al., 2010). In this study, *Cytospora rosicola* was also associated with canker disease of *Rosa* sp. in Tibet. *Cytospora rosicola* can be distinguished from *C. cincta* by having smaller and wider conidia (4.5–5.0 × 1–2 vs. 4.5–6.7 × 0.9–1.2), the conspicuous conceptacle, and flask-shaped conidiomata (Mehrabi et al., 2011). Furthermore, this species differs from *C. xinjiangensis* by conidia size (4.5–5.0 × 1–2 vs. 4.0–4.5 × 1–1.5 μm). Phylogenetically, we treat this species as new, which formed a separate branch. This species needs to be re-collected from *Rosa* sp. in Tibet of China, as presently no living culture is available.

**FIGURE 12:**
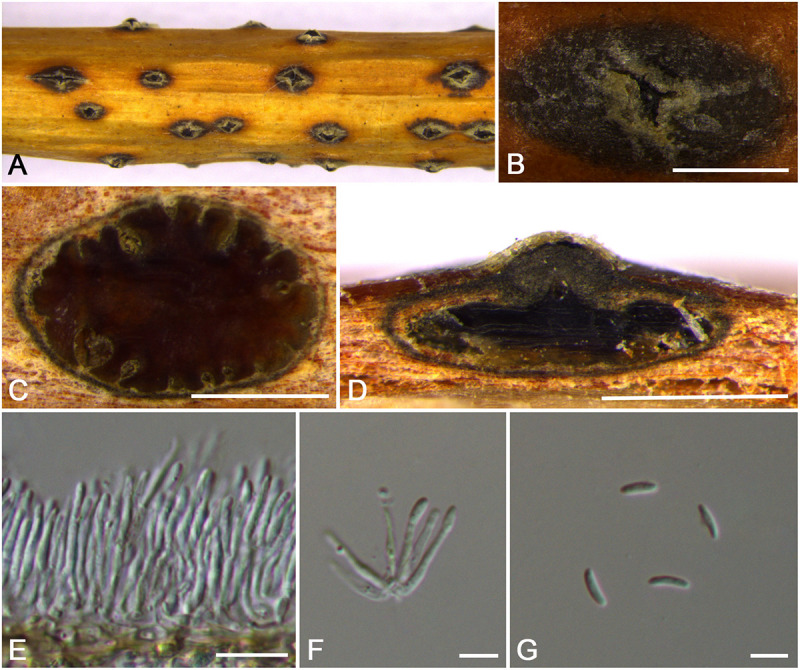
Morphology of *Cytospora sorbina* from *Sorbus tianschanica* (CF 20197660). **(A,B)** Habit of conidiomata on twig. **(C)** Transverse section of conidioma. **(D)** Longitudinal section through conidioma. **(E)** Conidiophores and conidiogenous cells. **(F,G)** Conidia. Bars: **(B–D)** = 500 μm; **(E)** = 10 μm; **(F,G)** = 5 μm.

***Cytospora sorbina*** M. Pan & X.L. Fan, sp. nov. (Figure 12)

MycoBank MB 834854

*Typification*: China. Xinjiang Uygur Autonomous Region: Bole Mongol Autonomous Prefecture, 44°46′13.44′′N, 81°13′58.72′′E, from branches of *Sorbus tianschanica*, 20 July 2017, *C.M. Tian* & *X.L. Fan* (**holotype** CF 20197660).

*Etymology*: Named after the host genus on which it was collected, *Sorbus tianschanica*.

*Descriptions*: Symptoms appeared as elongate and ovoid, orange or dark brown, raised, dehiscent lesions on the twigs or branches, with slightly discolored bark above the infected cambium. Asexual morph: Conidiomata pycnidial, immersed in bark, erumpent through the surface of bark when mature, erumpent, discoid to conical, with multiple locules, 740–1,120 μm ($\bar{x}$ = 910 μm, *n* = 30) in diam. Conceptacle dark. Ectostromatic disc yellow to orange, circular to ovoid, disc gray white to dark brown, 610–1,050 μm ($\bar{x}$ = 940 μm, *n* = 30) in diam. Ostiole conspicuous, circular, dark brown to black, 125–280 μm ($\bar{x}$ = 170 μm, *n* = 30) in diam. Locules numerous, irregular arrangement with common walls. Conidiophores hyaline, unbranched at base or occasionally branched. Conidiogenous cells enteroblastic, phialidic, sub-cylindrical to cylindrical. Conidia hyaline, smooth-walled, elongate-allantoid, aseptate, (4.0–)4.5–5.5 × 1–1.5 μm ($\bar{x}$ = 5.0 × 1.3 μm, *n* = 50). Sexual morph: not observed.

*Habitat and distribution*: Known only on *Sorbus tianschanica* from the type locality.

*Notes*: *Cytospora* species associated with *Sorbus* sp. were reported in previous studies such as *C. ampolliformis*, *C. leucostoma*, *C. populinopsis*, *C. sorbi*, and *C. sorbicola* (Norphanphoun et al., 2017; Fan et al., 2020). In this study, *C. sorbina* and *C. olivacea* are also reported from *Sorbus* sp. Morphologically, *C. sorbina* can be distinguished from *C. olivacea* by the common walls of its locules, as well as the larger conidia (4.5–5.5 × 1–1.5 vs. 4.0–5.0 × 1–1.5 μm). Based on phylogenetic analyses, this species forms separate lineages within the genus *Cytospora* and sister clade to *C. ochreae* and *C. japonica* (Supplementary Figure S1 and Figure 3).

**FIGURE 13:**
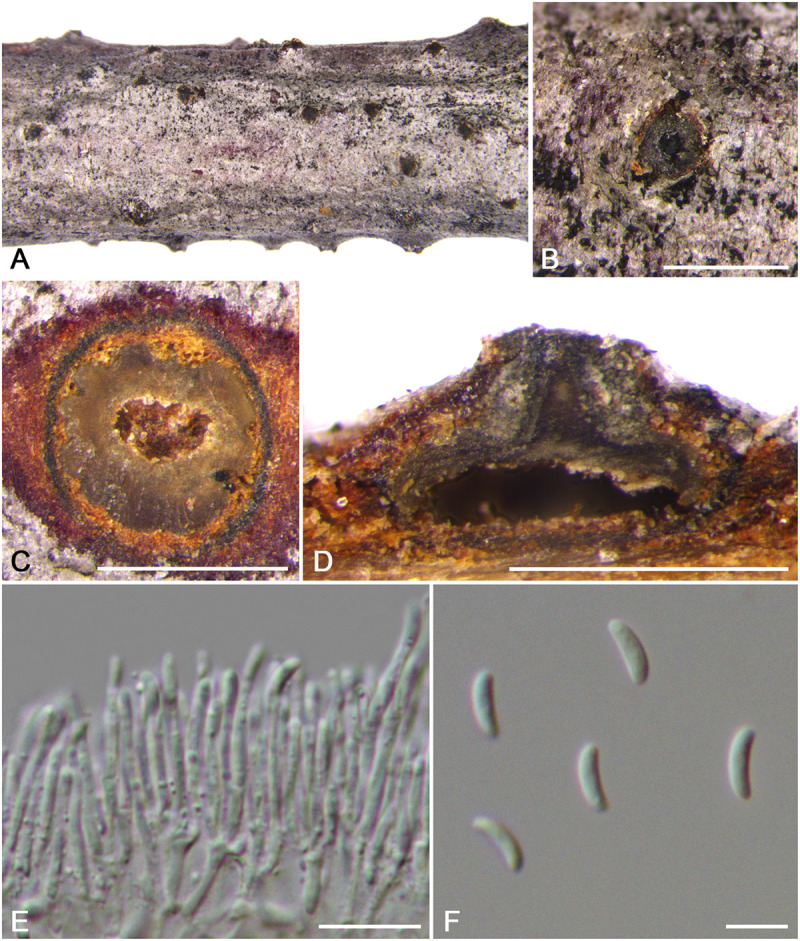
Morphology of *Cytospora tibetensis* from *Cotoneaster* sp. (CF 20197032). **(A,B)** Habit of conidiomata on twig. **(C)** Transverse section of conidioma. **(D)** Longitudinal section through conidioma. **(E)** Conidiophores and conidiogenous cells. **(F)** Conidia. Bars: **(B–D)** = 500 μm; **(E)** = 10 μm; **(F)** = 5 μm.

***Cytospora tibetensis*** M. Pan & X.L. Fan, sp. nov. (Figure 13)

MycoBank MB 834855

*Typification*: China. The Tibet Autonomous Region: Nyingchi City, Chayu County, Pineapple Village, 28°56′47.57″N, 97°25′31.93″E, from branches of *Cotoneaster* sp., 30 July 2016, *C.M. Tian* & *X.L. Fan* (**holotype** CF 20197032).

*Etymology*: Named after the geographical origin of the type strain, Tibet.

*Descriptions*: Symptoms appeared as circular and ovoid, dark brown or dark, raised, dehiscent lesions on the twigs or branches. Asexual morph: Conidiomata pycnidial, immersed in bark, erumpent through the surface of bark when mature, erumpent, discoid to conical, 585–650 μm ($\bar{x}$ = 610 μm, *n* = 30) in diam. Conceptacle conspicuous. Ectostromatic disc dark brown to gray, circular to ovoid, disc yellow to brown, 270–320 μm ($\bar{x}$ = 300 μm, *n* = 30) in diam. Ostiole conspicuous, one ostiole per disc, circular to ovoid, dark brown at the same level as the disc surface, 90–120 μm ($\bar{x}$ = 105 μm, *n* = 30) in diam. Locule undivided, circular to ovoid. Conidiophores hyaline, branched at base or occasionally not branched. Conidiogenous cells enteroblastic, phialidic, sub-cylindrical to cylindrical. Conidia hyaline, eguttulate, elongate-allantoid, aseptate, 5.0–5.5(–6.0) × 1.5–2 μm ($\bar{x}$ = 5.3 × 1.6 μm, *n* = 50). Sexual morph: not observed.

*Habitat and distribution*: Known only on *Cotoneaster* sp. from the type locality.

*Additional materials examined*: China. The Tibet Autonomous Region: Nyingchi City, Chayu County, Pineapple Village, 28°57′12.91″N, 97°25′20.26″E, from branches of *Cotoneaster* sp., 30 July 2016, *C.M. Tian* & *X.L. Fan* (CF 20197026); Nyingchi City, Chayu County, Pineapple Village, 28°56′47.46″N, 97°25′32.02″E, from branches of *Cotoneaster* sp., 30 July 2016, *C.M. Tian* & *X.L. Fan* (CF 20197029).

*Notes*: *Cytospora tibetensis* and *C. cotoneastricola* were founded on *Cotoneaster* sp., with the character of black conceptacle and single ostiole. However, *C. tibetensis* (5.0–5.5 × 1.5–2 μm) differs from *C. cotoneastricola* (13.0–14.0 × 2.5–3 μm) in having undivided locule with smaller conidia. Phylogenetic analyses based of combined six sequences data indicates that these species form two single lineages, separate from each other with high bootstrap support (Supplementary Figure S1 and Figure 3). *Cytospora tibetensis* is thus here considered as a novel species. This species needs to be re-collected from *Cotoneaster* sp. in Tibet of China, as presently no living culture is available.

**FIGURE 14:**
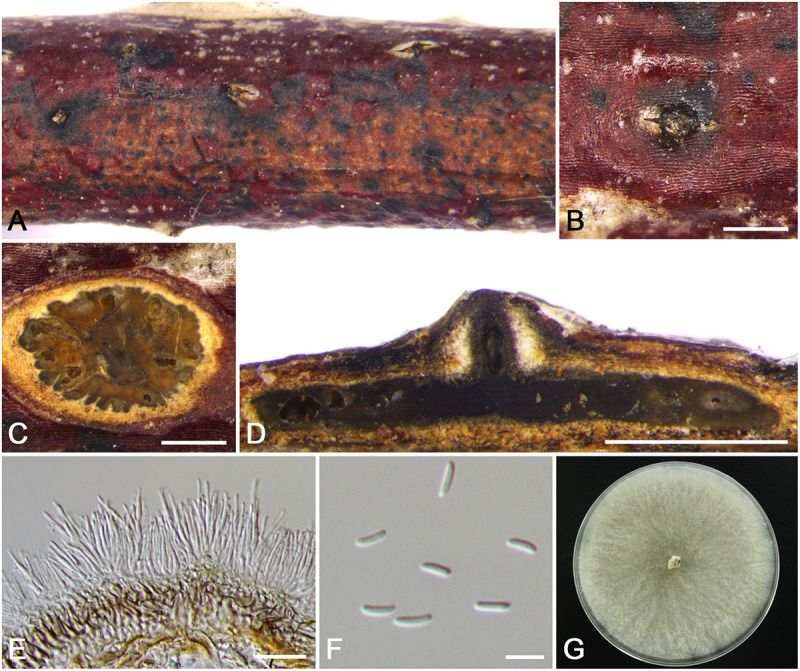
Morphology of *Cytospora xinjiangensis* from *Rosa* sp. (CF 20197520). **(A,B)** Habit of conidiomata on twig. **(C)** Transverse section of conidioma. **(D)** Longitudinal section through conidioma. **(E)** Conidiophores and conidiogenous cells. **(F)** Conidia. **(G)** Colonies on PDA for 2 weeks. Bars: **(B–D)** = 500 μm; **(E)** = 10 μm; **(F)** = 5 μm.

***Cytospora xinjiangensis*** M. Pan & X.L. Fan, sp. nov. (Figure 14)

MycoBank MB 834852

*Typification*: China. Xinjiang Uygur Autonomous Region: Ili Kazak Autonomous Prefecture, 45°98′54.00″N, 82°67′01.51″E, from branches of *Rosa* sp., 15 July 2017, *C.M. Tian* & *X.L. Fan* (**holotype** CF 20197520), ex-type living culture CFCC 53183.

*Etymology*: Named after the geographical origin of the type strain, Xinjiang.

*Descriptions*: Symptoms appeared as elongate or circular, dark, slightly sunken, dehiscent lesions on the twigs or branches, with discolored bark above the infected cambium. Asexual morph: Conidiomata pycnidial, immersed in bark, erumpent through the surface of bark when mature, erumpent, discoid, with large multiple locules, 965–1,410 μm ($\bar{x}$ = 1,145 μm, *n* = 30) in diam. Conceptacle absent. Ectostromatic disc dark brown to black, circular to ovoid, disc dark brown, 340–610 μm ($\bar{x}$ = 480 μm, *n* = 30) in diam. Ostiole unconspicuous, circular to ovoid, gray to black at the same level as the disc surface. Locule multiple and complex, subdivided by invaginations with common walls. Conidiophores hyaline, branched at base or occasionally not branched. Conidiogenous cells enteroblastic, phialidic, sub-cylindrical to cylindrical. Conidia hyaline, eguttulate, elongate-allantoid, aseptate, (3.5–)4.0–4.5(–5.0) × 1–1.5 ($\bar{x}$ = 4.3 × 1.3 μm, *n* = 50). Sexual morph: not observed.

*Culture characteristics*: Cultures on PDA are initially white, growing up to 7.5 cm after 3 days, becoming pale yellow after 14 days, while white are main. Colonies are flat with a uniform texture and becoming helcoid after 30 days. Sterile.

*Habitat and distribution*: Known only on *Rosa* sp. from the type locality and an additional locality in Xinjiang Uygur Autonomous Region.

*Additional material examined*: China. Xinjiang Uygur Autonomous Region: Bole Mongol Autonomous Prefecture, 44°48′47.29′′N, 81°06′22.07′′E, from branches of *Rosa* sp., 20 July 2017, *C.M. Tian* & *X.L. Fan* (CF 20197643), living culture CFCC 53182.

*Notes*: *Cytospora xinjiangensis* is associated with canker disease of *Rosa* sp., which has the same host with *C. leucostoma* and *C. rosicola*. Morphologically, *C. xinjiangensis* has unconspicuous ostiole with small conidia (4.0–4.5 × 1–1.5 μm), compared to the larger conidia of *C. leucostoma* (5.4–6.4 × 1.2–1.4 μm) (Norphanphoun et al., 2017) and wider conidia of *C. rosicola* (4.5–5.0 × 1–2 μm). The multigene phylogenetic analyses supported this species as a new species with high support values (MP/ML/BI = 100/99/1) (Figure 3).

## Other Species Reported From China

***Cytospora ceratosperma*** (Tode) G.C. Adams & Rossman, IMA Fungus, 6: 147, 2015.

*Basionym*: *Sphaeria ceratosperma* Tode, Fung. Mecklenb. Sel. (Lüneburg)2: 53. 1791.

*Synonyms*: *Sphaeria* sacculus Schwein., Schr. Naturf. Ges. Leipzig 1: 26. 1822.

*Valsa ceratosperma* (Tode) Maire, Publ. Inst. Bot. 3(4): 20. 1937.

*Cytospora sacculus* (Schwein.) Gvrit., Mikol. Fitopatol. 3: 207. 1969.

*Notes*: *Cytospora ceratosperma* has been reported in China from twigs and branches of *Malus pumila* and *Pyrus* sp. by Wang et al. (2007), which is regarded as a distinct species with multi-ostioles per disc, independent locule walls and grayish to yellow-brown and brownish gray colonies (Adams et al., 2005).

***Cytospora japonica*** (Miyabe & Hemmi) X.L. Fan, Persoonia, 45: 1–45, 2020.

*Basionym*: *Valsa japonica* Miyabe & Hemmi, J. Coll. Agric., Imp. Univ. Sapporo 7(4): 296, 1917.

*Notes*: *Cytospora japonica* has been reported in China from twigs and branches of *Prunus cerasifera* by Fan et al. (2020). This species was introduced as a common pathogen in Rosaceae host (Tai, 1979). It is characterized by discoid to conoid conidiomata with hyaline, allantoid, aseptate conidia (6.5–8.5 × 1.5–2 μm) as well as numerous locules, which arranged circularly or irregularly with common walls (Fan et al., 2020).

***Cytospora leucosperma*** (Pers.) Fr., Syst. Mycol. 2: 543, 1823.

*Basionym*: *Naemaspora leucosperma* Pers., Observ. Mycol. 1: 81, 1796.

*Synonyms*: *Sphaeria ambiens* Pers., Syn. Meth. Fung. 1: 44, 1801.

*Valsa ambiens* (Pers.) Fr., Summa Veg. Scand., Sectio Post. (Stockholm): 412, 1849.

*Cytospora ambiens* Sacc., Michelia 1(5): 519, 1879.

*Notes*: *Cytospora leucosperma* was chiefly isolated and recorded from *Pyrus* spp. in China (Teng, 1963; Tai, 1979; Zhuang, 2005). Infected branches collected from *Tilia* were regarded as the neotype (Urban, 1957; Spielman, 1985), but no living culture and DNA sequence data are available at present. *C. leucosperma* is similar to *C. mali* from *Malus* spp., leading to confusion in both morphology and molecular data (Wang et al., 2011).

***Cytospora mali*** Grove, British Stem- and Leaf-Fungi (Coelomycetes) (Cambridge) 1: 279, 1935.

*Synonyms*: *Valsa mali* Miyabe & G. Yamada, M. Miura Agr. Exp. Stn Bull. 4: 17, 1915.

*Notes*: *Cytospora mali* was chiefly isolated and discovered from apple (Teng, 1963; Tai, 1979; Wei, 1979; Zhuang, 2005; Wang et al., 2011). It has a similar morphology and close position in phylogeny with *C. leucosperma* (Fan et al., 2020). *Cytospora mali* can be distinguished from *C. leucosperma* by smaller conidiophores (7.5–15 × 1.5 vs. 17–25 × 2–2.5 μm). Furthermore, *C. leucosperma* is mostly isolated from *Pyrus* spp. (Fan et al., 2020).

***Cytospora populinopsis*** X.L. Fan & C.M. Tian, Persoonia, 45: 1–45, 2020.

*Notes*: *Cytospora populinopsis* was described by Fan et al. (2020) associated with canker disease of *Prunus salicina* and *Sorbus aucuparia* in China. It is characterized by having asci with four ascospores, which was similar with *C. populina* regarded as the pathogen for poplar canker (Fan et al., 2015b). *Cytospora populinopsis* differs from *C. populina* based on larger ascospores (13–21 × 2.5–5 vs. 12–13 × 3–4 μm) (Fan et al., 2015b, 2020).

***Cytospora rhodophila*** Sacc., Syll. Fung. (Abellini) 3: 253, 1884.

*Notes*: *Cytospora rhodophila* was recorded from *Rosa* sp. in China (Teng, 1963; Zhuang, 2005).

***Cytospora schulzeri*** Sacc. & P. Syd., Syll. Fung. (Abellini) 14(2): 918, 1899.

*Synonyms*: *Cytospora capitata* Schulzer & Sacc., Hedwigia 23: 109, 1884, non.

*Cytospora capitata* Fuckel, Reisen nach dem Nordpolarmeer 2: 34, 1874.

*Valsa malicola* Z. Urb., Èeská Mykol. 10: 209, 1956.

*Notes*: *Cytospora schulzeri* infected apple trees in China (Teng, 1963; Tai, 1979; Wei, 1979; Zhuang, 2005; Wang et al., 2011). This species differs from *C. mali*, which was also chiefly isolated and discovered from apple by having numerous ostioles and larger conidia (4.5–6.5 × 1–1.5 vs. 4–5 × 1 μm). In addition, *C. schulzeri* is distinguished from *C. parasitica* which is a common species associated with apple by its smaller conidia (4.5–6.5 × 1–1.5 vs. 6.5–8.0 × 1.3–1.5 μm) (Ariyawansa et al., 2015).

***Cytospora sibiraeae*** C.M. Tian et al., Fungal Diversity, 72: 44, 2015.

*Notes*: *Cytospora sibiraeae* is regarded as the pathogen responsible for the canker disease of *Sibiraea angustata* in China, which was reported by Liu et al. (2015). Pathogenic fungi in *Sibiraea* sp. are rarely reported.

***Cytospora spiraeae*** Fan, Phytotaxa, 338: 57, 2018.

*Notes*: *Cytospora spiraeae* was isolated from infected branches or twigs of *Spiraea salicifolia.* This species is similar with *C. schulzeri*, which has numerous ostioles and locules with common walls, but it can be distinguished by having smaller locules with a cenetr column compared with *C. schulzeri* (950–1,100 vs. 1,400–1,500 μm) (Zhu et al., 2018).

***Cytospora tamaricicola*** X.L. Fan & C.M. Tian, Persoonia, 45: 1–45, 2020.

*Notes*: *Cytospora tamaricicola* was described by Fan et al. (2020) associated with canker disease of *Rosa multiflora* and *Tamarix chinensis* in China. It is characterized by flask shaped to spherical perithecia with biseriate, elongate-allantoid, hyaline, aseptate ascospores (9–11.5 × 2–2.5 μm), pycnidia with multiple locules and thin-walled conidia (5.5–6 × 1–1.5 μm) (Fan et al., 2020).

**Key to *Cytospora* Species on *Prunus* spp. in China.**

1 Asexual morph present…………………………………………………….. 2
1 Asexual morph absent………………………………… *C. populinopsis*

2 Pycnidium without conceptacle……………………………………….. 3
2 Pycnidium with conceptacle…………………………………………….. 4

3 Locules with the common walls………………….. *C. pruni-mume*
3 Locules with the independent walls………………………………….. 5

4 Locules with the common walls…………………………. *C. japonica*
4 Locules with the independent walls………………………………….. 6

5 Size of conidia less than 6 μm…………………….. *C. cinnamomea*
5 Size of conidia more than 6 μm……………………….. *C. erumpens*

6 Sexual morph absent…………………………………………. *C. olivacea*
6 Sexual morph present…………………………………… *C. leucostoma*

**Key to *Cytospora* species on *Malus* spp. in China**

1 Sexual morph absent………………………………………………………… 2
1 Sexual and asexual morph present……………………………………. 3

2 Size of conidia less than 7 μm…………………………. *C. parasitica*
2 Size of conidia more than 7 μm……………… *C. mali-spectabilis*

3 Pycnidial stromata with single ostiole………………………………. 4
3 Pycnidial stromata with numerous ostioles………. *C. schulzeri*

4 Locules with the common walls………………………………. *C. mali*
4 Locules with the independent walls…………….. *C. ceratosperma*

## Discussion

In this study, we accepted 23 species of *Cytospora* from infected plants of Rosaceae in China, including 10 new species (*Cytospora cinnamomea*, *C. cotoneastricola*, *C. ochracea*, *C. olivacea*, *C. pruni-mume*, *C. rosicola*, *C. mali-spectabilis*, *C. sorbina*, *C. tibetensis*, and *C. xinjiangensis*), and 13 known taxa (*Cytospora ceratosperma*, *C. erumpens*, *C. japonica*, *C. leucosperma*, *C. leucostoma*, *C. mali*, *C. parasitica*, *C. populinopsis*, *C. rhodophila, C. schulzeri*, *C. sibiraeae*, *C. spiraeae*, and *C. tamaricicola*). The current study revealed the attempt to clarify the taxonomy of *Cytospora* species and extensive host distribution of Rosaceae in China.

The plants of Rosaceae are important ecological and economic tree species in China. However, the current study indicates that the incidence of *Cytospora* species is serious and have different symptoms in various hosts, including 20 host species of nine genera in Rosaceae, i.e., *Cotoneaster*, *Crataegus*, *Malus*, *Prunus*, *Pyrus*, *Rosa*, *Sibiraea*, *Sorbus*, and *Spiraea*. The result coincides with previous reports that widely extended *Cytospora* species have been identified to occur in many host species (Adams et al., 2005; Fan et al., 2014a, 2015a, b; Ariyawansa et al., 2015; Liu et al., 2015; Maharachchikumbura et al., 2015, 2016; Hyde et al., 2016; Li et al., 2016) in the current study. Six *Cytospora* species recovered from diverse *Prunus* species in California was reported by Lawrence et al. (2018), which are *Cytospora amygdali*, *C. californica*, *C. eucalypti*, *C. longispora*, *C. plurivora*, and *C. sorbicola*. The current results also supplement seven different *Cytospora* species afflicted *Prunus* host plants in China. The comparison shows that the species occurrence may be related by geographical and environmental factors, rather than the taxa actually being host specific. *Cytospora* species and accumulation of DNA dataset are required to expand our understanding of their host range and distribution. Furthermore, *Cytospora cotoneastricola*, *C. ochracea*, and *C. tibetensis* were all collected from *Cotoneaster* sp., which indicates that the same host could be infected by more than one species. Stevens (1919) summarized the symptoms and species of Rosaceae infected by *Cytospora*, whereas these reports lacked molecular data. Only a few relative taxonomic studies of Cytospora canker or dieback disease from the plants of Rosaceae were reported, such as *Cytospora chrysosperma, C. cincta, C. leucostoma*, and *C. schulzeri* (Mehrabi et al., 2011). Moreover, the host specificity and pathogenicity of many *Cytospora* species are poorly known. In the current study, *Cytospora leucostoma* is a common species associated with stem canker diseases of woody plants of Rosaceae, mainly Prunoideae host plants, and *C. mali*, *C. parastica*, and *C. schulzeri* are the common species collected from apple trees.

In China, *Cytospora* species from cankered apple and pear bark were examined and compared with morphology and ITS sequence data (Wang et al., 2007, 2011). The species identity of the pathogen of *Valsa* (now *Cytospora*) canker on pear tree was determined through a combined study of ITS sequence data and cultural characteristics of isolates from apple trees and pear trees in China (Zhang et al., 2007). Ma et al. (2018) clarified and illustrated *C. parasitica* from the *Malus* sp. using the ITS, LSU, and *tef1-*α regions. Fan et al. (2020) summarized 52 species of *Cytospora* and recommended the dataset of ITS, LSU *act*, *rpb2*, *tef1-*α, and *tub2* gene regions. At present, China is a hot place to study these taxa as many species of *Cytospora* are isolated from important hosts such as Rosaceae. Thus, further studies are required to discover the species of *Cytospora* in China.

## Data Availability Statement

The datasets generated for this study can be found in the MK672943–MK672956, MK672958–MK672985, MK672987–MK673009, MK673011–MK673039, MK673041–MK673069, MK673071–MK673099, and MK673101– MK673111.

## Author Contributions

All authors contributed extensively to the work presented in the manuscript. XF and CT conceived and designed the experiments. MP and HZ performed the experiments. MP, HZ, and GB analyzed the data. GB polished the language. MP wrote the manuscript. XF revised and approved the final version of the manuscript.

## Conflict of Interest

The authors declare that the research was conducted in the absence of any commercial or financial relationships that could be construed as a potential conflict of interest.

## Footnotes

1 https://raxml-ng.vital-it.ch

2 www.treebase.org
