# Supplementary material for: High Diversity of Cytospora Associated With Canker and Dieback of Rosaceae in China, With 10 New Species Described
Source: Front Plant Sci. 2020 Jul 3;11:690. doi: 10.3389/fpls.2020.00690 (PMC7350520; doi:10.3389/fpls.2020.00690)
Supplement: Supplementary file 1 [file Data_Sheet_1.PDF]

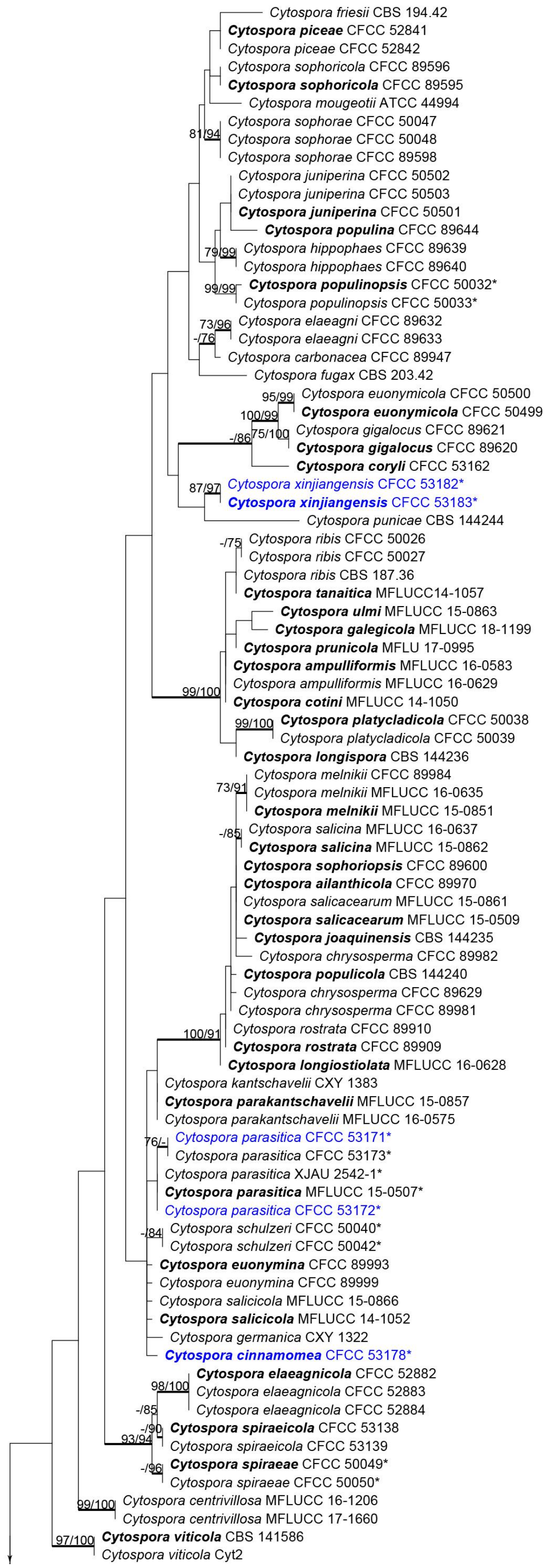
Cytospora friesii CBS 194.42
Cytospora piceae CFCC 52841
Cytospora piceae CFCC 52842
Cytospora sophoricola CFCC 89596
Cytospora sophoricola CFCC 89595
Cytospora mougeotii ATCC 44994
Cytospora sophorae CFCC 50047
Cytospora sophorae CFCC 50048
Cytospora sophorae CFCC 89598
Cytospora juniperina CFCC 50502
Cytospora juniperina CFCC 50503
Cytospora juniperina CFCC 50501
Cytospora populina CFCC 89644
Cytospora hippophaes CFCC 89639
Cytospora hippophaes CFCC 89640
Cytospora populinopsis CFCC 50032*
Cytospora populinopsis CFCC 50033*
Cytospora elaeagni CFCC 89632
Cytospora elaeagni CFCC 89633
Cytospora carbonacea CFCC 89947
Cytospora fugax CBS 203.42
Cytospora euonymicola CFCC 50500
Cytospora euonymicola CFCC 50499
Cytospora gigalocus CFCC 89621
Cytospora gigalocus CFCC 89620
Cytospora coryli CFCC 53162
Cytospora xinjiangensis CFCC 53182*
Cytospora xinjiangensis CFCC 53183*
Cytospora punicae CBS 144244
Cytospora ribis CFCC 50026
Cytospora ribis CFCC 50027
Cytospora ribis CBS 187.36
Cytospora tanaitica MFLUCC14-1057
Cytospora ulmi MFLUCC 15-0863
Cytospora galegicola MFLUCC 18-1199
Cytospora prunicola MFLU 17-0995
Cytospora ampulliformis MFLUCC 16-0583
Cytospora ampulliformis MFLUCC 16-0629
Cytospora cotini MFLUCC 14-1050
Cytospora platycladicola CFCC 50038
Cytospora platycladicola CFCC 50039
Cytospora longispora CBS 144236
Cytospora melnikii CFCC 89984
Cytospora melnikii MFLUCC 16-0635
Cytospora melnikii MFLUCC 15-0851
Cytospora salicina MFLUCC 16-0637
Cytospora salicina MFLUCC 15-0862
Cytospora sophoriopsis CFCC 89600
Cytospora ailanthicola CFCC 89970
Cytospora salicacearum MFLUCC 15-0861
Cytospora salicacearum MFLUCC 15-0509
Cytospora joaquinensis CBS 144235
Cytospora chrysosperma CFCC 89982
Cytospora populicola CBS 144240
Cytospora chrysosperma CFCC 89629
Cytospora chrysosperma CFCC 89981
Cytospora rostrata CFCC 89910
Cytospora rostrata CFCC 89909
Cytospora longiostiolata MFLUCC 16-0628
Cytospora kantschavelii CXY 1383
Cytospora parakantschavelii MFLUCC 15-0857
Cytospora parakantschavelii MFLUCC 16-0575
Cytospora parasitica CFCC 53171*
Cytospora parasitica CFCC 53173*
Cytospora parasitica XJAU 2542-1*
Cytospora parasitica MFLUCC 15-0507*
Cytospora parasitica CFCC 53172*
Cytospora schulzeri CFCC 50040*
Cytospora schulzeri CFCC 50042*
Cytospora euonymina CFCC 89993
Cytospora euonymina CFCC 89999
Cytospora salicicola MFLUCC 15-0866
Cytospora salicicola MFLUCC 14-1052
Cytospora germanica CXY 1322
Cytospora cinnamomea CFCC 53178*
Cytospora elaeagnicola CFCC 52882
Cytospora elaeagnicola CFCC 52883
Cytospora elaeagnicola CFCC 52884
Cytospora spiraeicola CFCC 53138
Cytospora spiraeicola CFCC 53139
Cytospora spiraeae CFCC 50049*
Cytospora spiraeae CFCC 50050*
Cytospora centrivillosa MFLUCC 16-1206
Cytospora centrivillosa MFLUCC 17-1660
Cytospora viticola CBS 141586
Cytospora viticola Cyt2

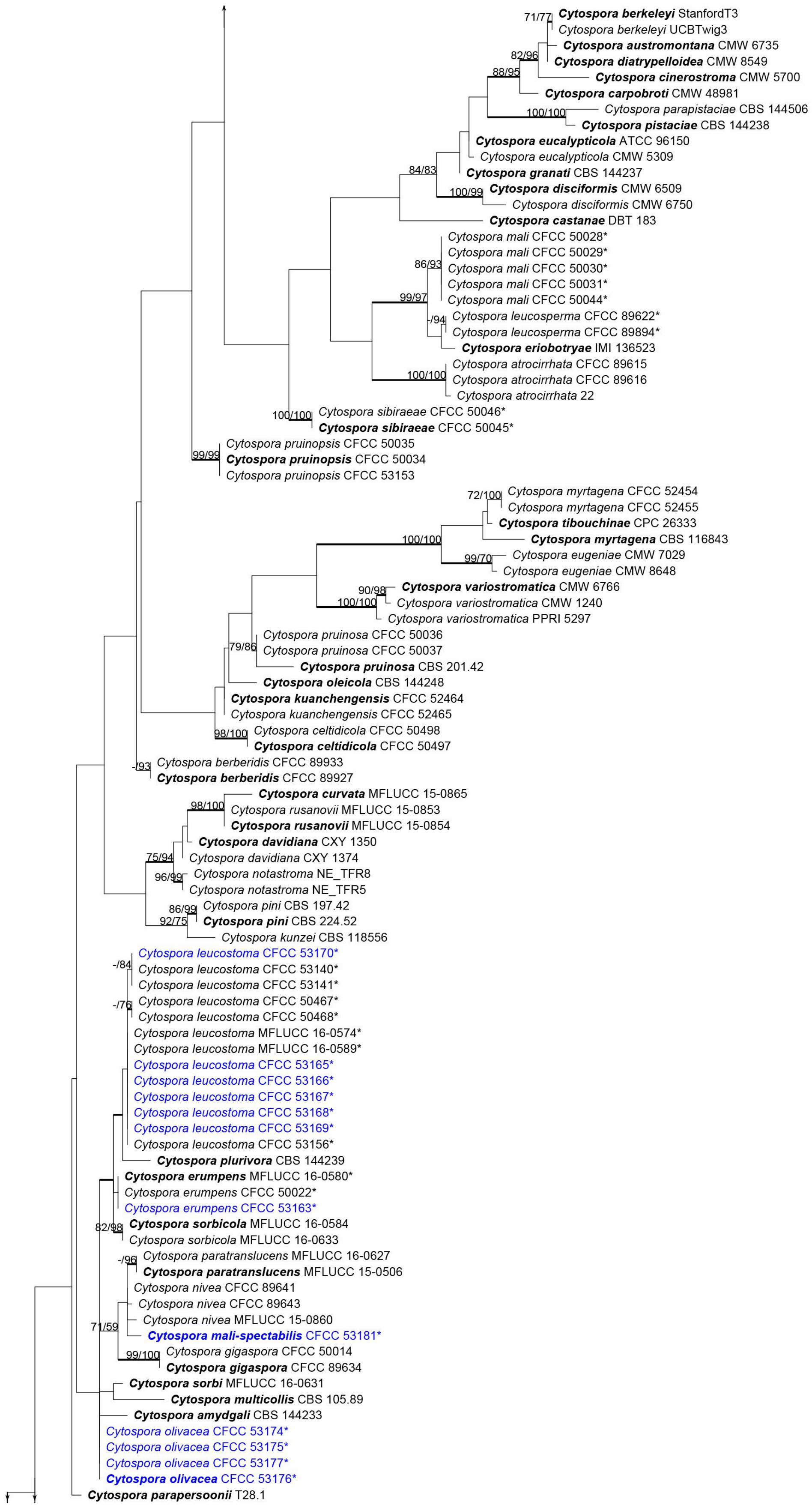
71/77
Cytospora berkeleyi StanfordT3
Cytospora berkeleyi UCBTwig3
Cytospora austromontana CMW 6735
82/96
Cytospora diatrypelloidea CMW 8549
88/95
Cytospora cinerostroma CMW 5700
Cytospora carpobroti CMW 48981
Cytospora parapistaciae CBS 144506
100/100
Cytospora pistaciae CBS 144238
Cytospora eucalypticola ATCC 96150
Cytospora eucalypticola CMW 5309
84/83
Cytospora granati CBS 144237
100/99
Cytospora disciformis CMW 6509
Cytospora disciformis CMW 6750
Cytospora castanae DBT 183
Cytospora mali CFCC 50028*
Cytospora mali CFCC 50029*
86/93
Cytospora mali CFCC 50030*
Cytospora mali CFCC 50031*
99/97
Cytospora mali CFCC 50044*
-/94
Cytospora leucosperma CFCC 89622*
Cytospora leucosperma CFCC 89894*
Cytospora eriobotryae IMI 136523
Cytospora atrocirrhata CFCC 89615
100/100
Cytospora atrocirrhata CFCC 89616
Cytospora atrocirrhata 22
100/100
Cytospora sibiraeae CFCC 50046*
Cytospora sibiraeae CFCC 50045*
Cytospora pruinopsis CFCC 50035
99/99
Cytospora pruinopsis CFCC 50034
Cytospora pruinopsis CFCC 53153
72/100
Cytospora myrtagena CFCC 52454
Cytospora myrtagena CFCC 52455
Cytospora tibouchinae CPC 26333
100/100
Cytospora myrtagena CBS 116843
99/70
Cytospora eugeniae CMW 7029
Cytospora eugeniae CMW 8648
90/98
Cytospora variostromatica CMW 6766
100/100
Cytospora variostromatica CMW 1240
Cytospora variostromatica PPRI 5297
Cytospora pruinosa CFCC 50036
79/86
Cytospora pruinosa CFCC 50037
Cytospora pruinosa CBS 201.42
Cytospora oleicola CBS 144248
Cytospora kuanchengensis CFCC 52464
Cytospora kuanchengensis CFCC 52465
98/100
Cytospora celtidicola CFCC 50498
Cytospora celtidicola CFCC 50497
Cytospora berberidis CFCC 89933
-/93
Cytospora berberidis CFCC 89927
Cytospora curvata MFLUCC 15-0865
98/100
Cytospora rusanovii MFLUCC 15-0853
Cytospora rusanovii MFLUCC 15-0854
Cytospora davidiana CXY 1350
75/94
Cytospora davidiana CXY 1374
96/99
Cytospora notastroma NE_TFR8
Cytospora notastroma NE_TFR5
86/99
Cytospora pini CBS 197.42
92/75
Cytospora pini CBS 224.52
Cytospora kunzei CBS 118556
Cytospora leucostoma CFCC 53170*
-/84
Cytospora leucostoma CFCC 53140*
Cytospora leucostoma CFCC 53141*
-/76
Cytospora leucostoma CFCC 50467*
Cytospora leucostoma CFCC 50468*
Cytospora leucostoma MFLUCC 16-0574*
Cytospora leucostoma MFLUCC 16-0589*
Cytospora leucostoma CFCC 53165*
Cytospora leucostoma CFCC 53166*
Cytospora leucostoma CFCC 53167*
Cytospora leucostoma CFCC 53168*
Cytospora leucostoma CFCC 53169*
Cytospora leucostoma CFCC 53156*
Cytospora plurivora CBS 144239
Cytospora erumpens MFLUCC 16-0580*
Cytospora erumpens CFCC 50022*
Cytospora erumpens CFCC 53163*
82/98
Cytospora sorbicola MFLUCC 16-0584
Cytospora sorbicola MFLUCC 16-0633
-/96
Cytospora paratranslucens MFLUCC 16-0627
Cytospora paratranslucens MFLUCC 15-0506
Cytospora nivea CFCC 89641
Cytospora nivea CFCC 89643
71/59
Cytospora nivea MFLUCC 15-0860
Cytospora mali-spectabilis CFCC 53181*
99/100
Cytospora gigaspora CFCC 50014
Cytospora gigaspora CFCC 89634
Cytospora sorbi MFLUCC 16-0631
Cytospora multicollis CBS 105.89
Cytospora amydgali CBS 144233
Cytospora olivacea CFCC 53174*
Cytospora olivacea CFCC 53175*
Cytospora olivacea CFCC 53177*
Cytospora olivacea CFCC 53176*
Cytospora parapersoonii T28.1

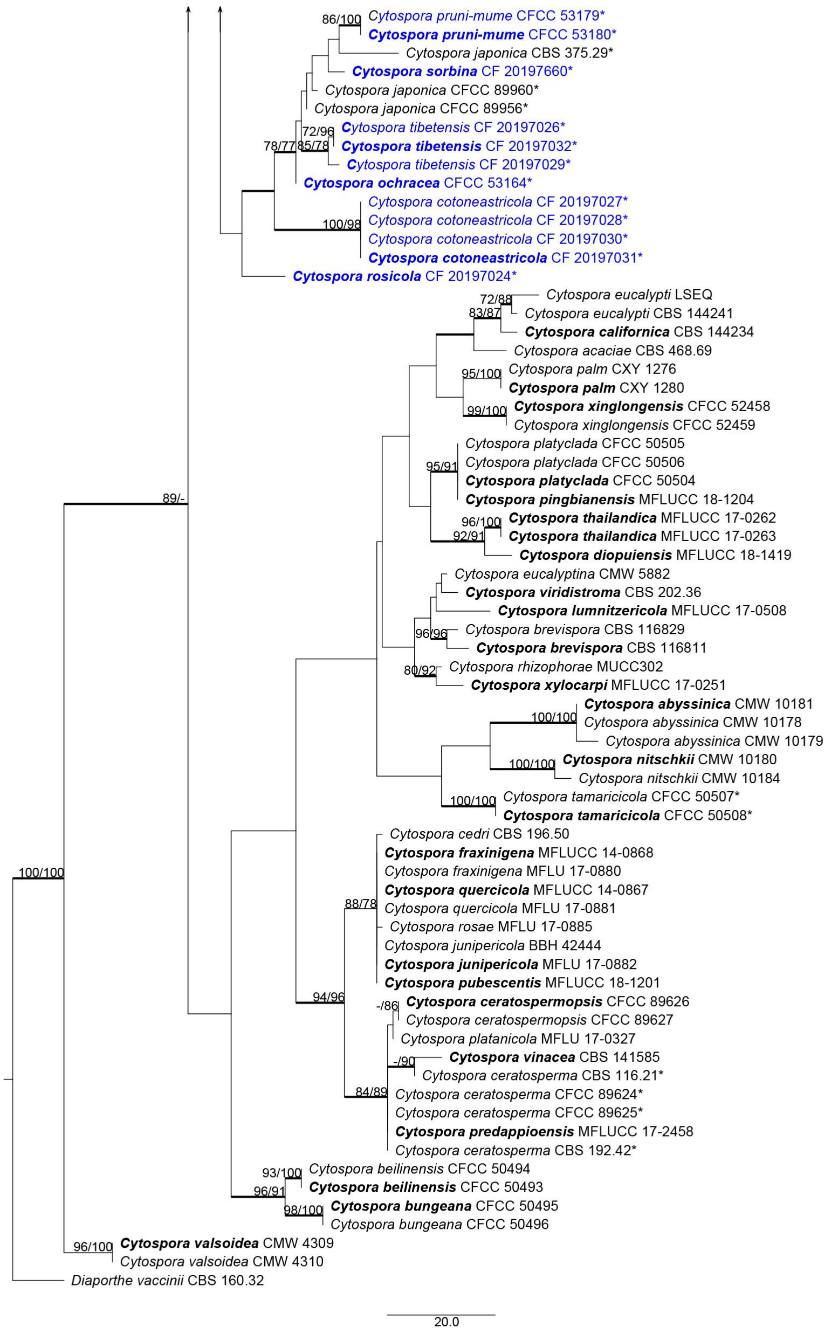

Figure S1. Phylogram of *Cytospora* based on ITS gene. MP and ML bootstrap support values above 70 % are shown at the first and second position. Thickened branches represent posterior probabilities above 0.95 from BI. Ex-type strains are in bold. Strains in current study are in blue. All the *Cytospora* species listed from Rosaceae plants in China are marked with *.
